# Strain-Induced Photochemical Opening of Ferrocene[6]cycloparaphenylene: Uncaging of Fe^2+^ with Green Light

**DOI:** 10.1021/jacs.4c15818

**Published:** 2025-01-17

**Authors:** Remigiusz B. Kręcijasz, Juraj Malinčík, Simon Mathew, Peter Štacko, Tomáš Šolomek

**Affiliations:** † Van ‘t Hoff Institute for Molecular Sciences, University of Amsterdam, 1098 XH Amsterdam, Netherlands; ‡ Department of Chemistry, University of Zurich, CH-8057 Zurich, Switzerland

## Abstract

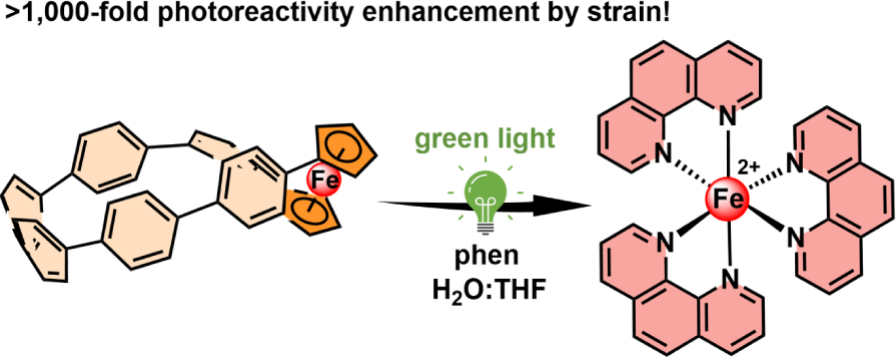

We present the synthesis, structural analysis, and remarkable reactivity of the first carbon nanohoop that fully incorporates ferrocene in the macrocyclic backbone. The high strain imposed on the ferrocene by the curved nanohoop structure enables unprecedented photochemical reactivity of this otherwise photochemically inert metallocene complex. Visible light activation triggers a ring-opening of the nanohoop structure, fully dissociating the Fe–cyclopentadienyl bonds in the presence of 1,10-phenanthroline. This process uncages Fe^2+^ ions captured in the form of [Fe(phen)_3_]^2+^ complex in high chemical yield and can operate efficiently in a water-rich solvent with green light excitation. The measured quantum yields of [Fe(phen)_3_]^2+^ formation show that embedding ferrocene into a strained nanohoop boosts its photoreactivity by 3 orders of magnitude compared to an unstrained ferrocene macrocycle or ferrocene itself. Our data suggest that the dissociation occurs by intercepting the photoexcited triplet state of the nanohoop by a nucleophilic solvent or external ligand. The strategy portrayed in this work proposes that new, tunable reactivity of analogous metallamacrocycles can be achieved with spatial and temporal control, which will aid and abet the development of responsive materials for metal ion delivery and supramolecular, organometallic, or polymer chemistry.

## Introduction

Iron is the single most important transition metal in the human body. While primarily renowned for its role in oxygen transport and storage within hemoglobin and myoglobin,^1^ iron fulfills numerous other critical roles in biological systems.^2^ Iron is a crucial component of cytochromes involved in the energy-providing electron transport chain in mitochondria.^3^ Ribonucleotide reductase—an iron-dependent enzyme—is necessary in the synthesis of deoxyribonucleotides, the building blocks of DNA that are crucial for cell division and repair.^4^ Iron also acts as a cofactor for various enzymes responsible for protection from oxidative stress^5,6^ or in the synthesis/degradation of hormones and neurotransmitters.^7−9^ Nature has developed a sophisticated system to tightly regulate iron’s uptake and homeostasis through proteins and hormones like transferrin, ferroportin, ferritin, and hepcidin.^10^ This system ensures a delicate balance between meeting physiological iron needs and preventing the adverse effects of iron overload. For instance, iron levels are reduced to restrict its availability to pathogens in response to inflammation, but this action can also impair immune cell function.^11−13^ Artificial systems that mimic these functions or exert spatiotemporal control over iron levels are consequently attractive in the context of potential biomedical applications.

Ferrocene (**Fc**) with its captivating sandwich structure featuring a central iron atom represents one of the most studied organometallic compounds since its discovery in the early 1950s.^14^ Its popularity can be attributed to a unique combination of its redox chemistry, structural fluxionality, and exceptional chemical and photochemical stability that parallels those of aromatic compounds. In fact, the iron–cyclopentadienyl (Fe–Cp) bond dissociation energy (BDE) of ∼90 kcal mol^–1^ is similar to that of a typical covalent C–C bond.^15^ As a result, the excellent redox properties and robustness made this metallocene class of materials particularly attractive for diverse applications that span polymer science,^16,17^ sensing,^18,19^ catalysis,^18,20^ biochemistry,^21^ and molecular electronics.^18,22^

**Figure 1:**
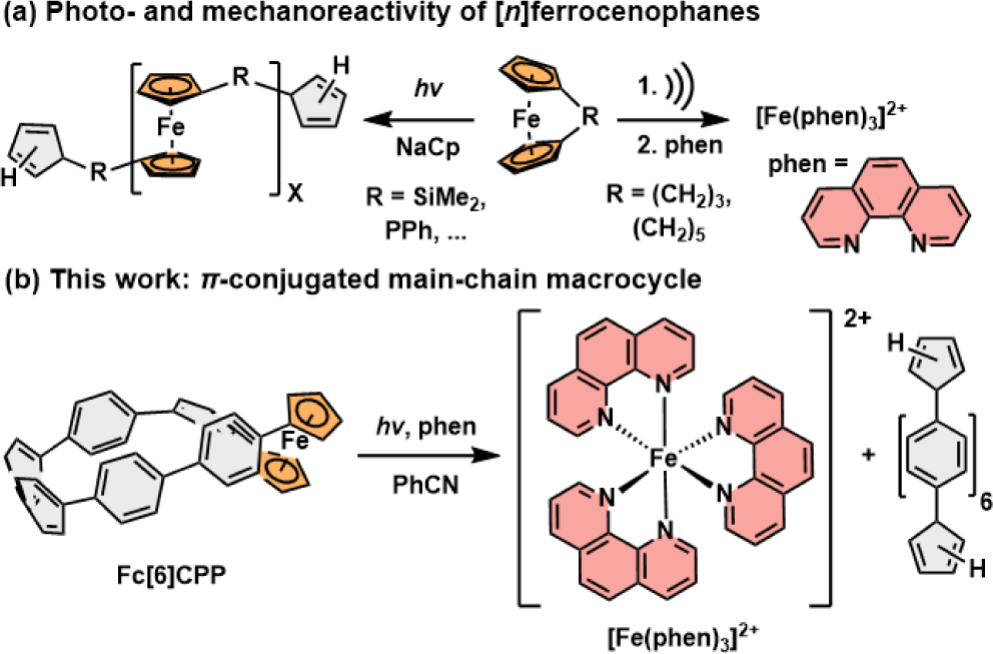
(a) Examples of photo-^16,23−27^ and mechanoactive^28−30^ [*n*]ferrocenophanes, and (b) conceptually new photoactive ferrocene-based π-conjugated macrocycle described in this work.

Imparting strain to **Fc** increases the propensity of this otherwise inert compound to undergo cleavage of the Fe–Cp bond. This has been successfully exploited in a light-induced ring-opening polymerization (ROP) via Fe–Cp bond dissociation in strained [*n*]ferrocenophanes (*E*_strain_ = 14–31 kcal mol^–1^).^16,23−27^ In these molecules, the two Cp rings are bridged via a few atoms (*n* = 1–2, Figure 1a) linked with single bonds. Although diverse polymers can be achieved via substitution of the bridging atoms or the Fc core itself, the linkers must be short, and they interrupt the π-conjugation in the polymer. Recently, polymers with **Fc** incorporated in their backbone were shown to be susceptible to mechanically triggered Fe–Cp bond scission (Figure 1a), eventually releasing Fe^2+^ or Fe^3+^ ions.^28−30^ Here, [*n*]ferrocenophanes (*n* = 3, 5) were also used to tune the mechanical sensitivity of the Fc mechanophore.^29^

Achieving iron release on demand using benign light activation would represent a great tool to control the concentration of available iron in a sample. However, such accomplishment would require developing robust methods to tune the strain in the Fc unit to control its reactivity. Cycloparaphenylenes (CPPs)^31,32^ are a unique class of highly strained, curved π-conjugated macrocycles consisting solely of phenylene rings connected through *para* positions. CPPs and related carbon-rich molecular systems, so-called carbon nanohoops, can be modified to alter the molecular strain and curvature by manipulating the number of *para*-phenylene units in the macrocycle. Therefore, the CPP scaffold offers an elegant way to control the structural strain of a unit incorporated in the corresponding carbon nanohoop. We hypothesized that embedding **Fc** into a highly strained, fully π-conjugated macrocycle^33,34^ such as CPP via both Cp rings could represent a robust strategy to impose the strain on **Fc**, enabling the control of **Fc** reactivity. The resultant conjugated, shape-persistent metallocene carbon nanohoops could lead to new applications demanding light- and force-sensitive materials. Although a handful of organometallic compounds based on the CPP scaffold have been reported, the metal atom in these structures is not an integral part of the macrocyclic backbone and it is therefore subject to a lower amount of strain than what the curved CPP structures could provide.^33−38^

Here, we report the synthesis and properties of the first ferrocene-cycloparaphenylene **Fc[*****n*****]CPP** (*n* = 6) with Fc enclosed in a loop of six *para*-phenylene rings. The considerable strain imparted on the Fc unit in **Fc[6]CPP** enables its unprecedented photoreactivity that allows to open the nanohoop structure and release Fe^2+^ in high yield at ambient conditions using benign blue or green light in polar solvents (Figure 1b). The nanohoop **Fc[6]CPP** thus serves as a photoactivatable molecular storage system of Fe^2+^ ions, reminiscent of ferritin but with deliberate spatiotemporal control.

## Results and Discussion

**Scheme 1:**
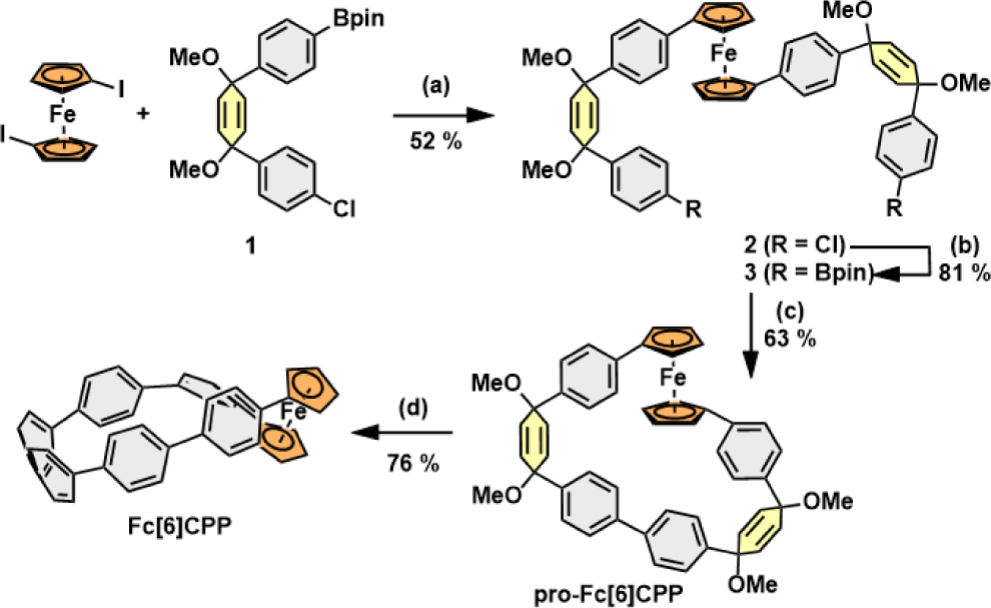
Synthesis of **Fc[6]CPP** Conditions Reaction conditions: (a) **1** (3 equiv), Pd(dppf)Cl_2_ (0.05 equiv), NaOH (4 equiv), DME/H_2_O, 85 °C, 20 h; (b) B_2_pin_2_ (4 equiv), Pd_2_(dba)_3_ (0.05 equiv), XPhos (0.2 equiv), KOAc (8 equiv), 1,4-dioxane, 110 °C, 16 h; (c) Pd(dppf)Cl_2_ (0.1 equiv), KF (1 equiv), B(OH)_3_ (5 equiv), THF/H_2_O, air, 40 °C, 20 h; and (d) H_2_SnCl_4_ (3.6 equiv), THF, RT, 2 h.

**Fc[6]CPP** was prepared in four steps (Scheme 1) from the reported 1,1′-diiodoferrocene and building block **1** following the methodology developed by Jasti.^31^ First, the Suzuki cross-coupling of 1,1′-diiodoferrocene with **1** provided intermediate **2** in a 52% yield. The chlorides in intermediate **2** were then replaced by Miyaura borylation forming diboronate **3** in 81% yield. Subsequently, the intramolecular oxidative homocoupling^39^ of **3** afforded the pro-aromatic macrocycle **pro-Fc[6]CPP** in a very good 63% yield. The reductive aromatization of the two cyclohexa-2,5-dienyl units in **pro-Fc[6]CPP** using SnCl_2_/HCl^40^ proceeded smoothly, and a pure sample of **Fc[6]CPP** could be isolated avoiding column chromatography in 76% yield. The final nanohoop **Fc[6]CPP** is soluble in dichloromethane and THF and displays high chemical stability when stored under ambient conditions over a few months as a solid. The structures of both macrocycles **pro-Fc[6]CPP** and **Fc[6]CPP** were confirmed by 1D and 2D NMR spectroscopy and high-resolution mass spectrometry.

**Figure 2:**
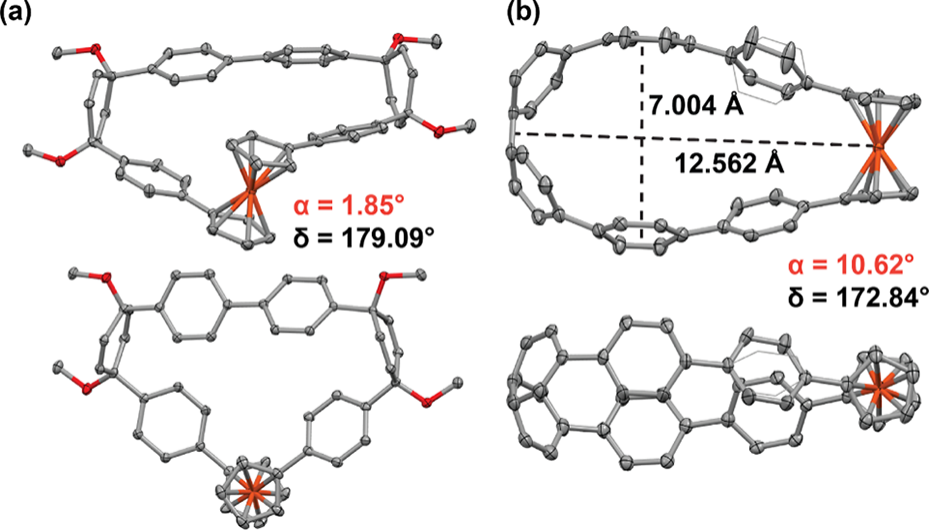
X-ray crystal structures of (a) **pro-Fc[6]CPP** (selected conformer, see Supporting Information) and (b) **Fc[6]CPP** (thermal ellipsoids shown at 50% probability; all hydrogen atoms and solvent were omitted for clarity). The carbon atoms of one phenyl group in (b) are disordered over two sites with relative occupancies of 0.602:0.398.

Single crystals were obtained by vapor diffusion of *n*-hexane into a toluene solution (20 °C) of **pro-Fc[6]CPP** and vapor diffusion of methanol into a THF solution (at 4 °C) of **Fc[6]CPP**. The X-ray diffraction analysis unequivocally confirmed their macrocyclic structure (Figure 2). Compound **pro-Fc[6]CPP** crystallized in the triclinic *P*–1 space group, and Fc fluxionality allowed it to adopt a triangular shape. Both enantiomers with opposite helicity (Figures S2 and S3) can be clearly distinguished in the crystal. Nanohoop **Fc[6]CPP** crystallized in the monoclinic *P*2_1_ space group. Here, the Fc flexibility allowed the macrocycle to adopt an oval shape (Figure 2b) typical for *meta*-CPPs^41^ and related nanohoops.^42,43^ The size of the elliptic cavity is 12.6 Å in length and 7.0 Å in width. The connecting Cp carbon atoms in the Fc moiety are nearly eclipsed. Further analysis of the crystal structures revealed the effect of the strain imparted to Fc by the curvature of the macrocycles. The tilt angle α defined by the planes of the Cp rings and the Cp–Fe–Cp angle δ—common descriptors describing ferrocenophanes (see Figure S1)—differ in **pro-Fc[6]CPP** (Table S2) from the ideal values^44^ in unstrained **Fc** (α = 0°; δ = 180°) only slightly. On the other hand, the values determined for **Fc[6]CPP** (10.62°; 172.84°) clearly indicate that part of the total strain in the macrocycle has been transferred to Fc. The deviation from the ideal angles correlates with *E*_strain_ values of 13.7 and 82.6 kcal mol^–1^ calculated for both **pro-Fc[6]CPP** and **Fc[6]CPP**, respectively, using homodesmotic reactions (Scheme S1). The DFT-calculated geometries reproduce the crystal structures well, and the calculated strain in **Fc[6]CPP** approaches that of [7]CPP (*E*_strain_ = 84.0 kcal mol^–1^).^45^ Despite the significant strain, the Fc in **Fc[6]CPP** is markedly less distorted than less strained [1]- and [2]ferrocenophanes (14–31 kcal mol^–1^; α = 19–31° and δ = 156–167°).^26,46−48^ Although the strain energies in [*n*]ferrocenophanes and **Fc[6]CPP** markedly differ, the strain calculated per atom is comparable, which highlights its different distribution. The values of α and δ suggest that Fc in **Fc[6]CPP** is less strained than that in the known [*n*]ferrocenophanes. The large distortion of the Fc moiety in the latter compounds is known to weaken the Fe–Cp bond and to induce its cleavage upon irradiation. However, the Fc distortion observed for **Fc[6]CPP** matches that found in Fe(η-C_5_H_4_)_2_(CHCHCHCH) (10.2°; 173.08°),^49^ a compound known to be stable in air as a solid and in a solution,^50^ for which no photolytic processes are reported in the literature.

**Figure 3:**
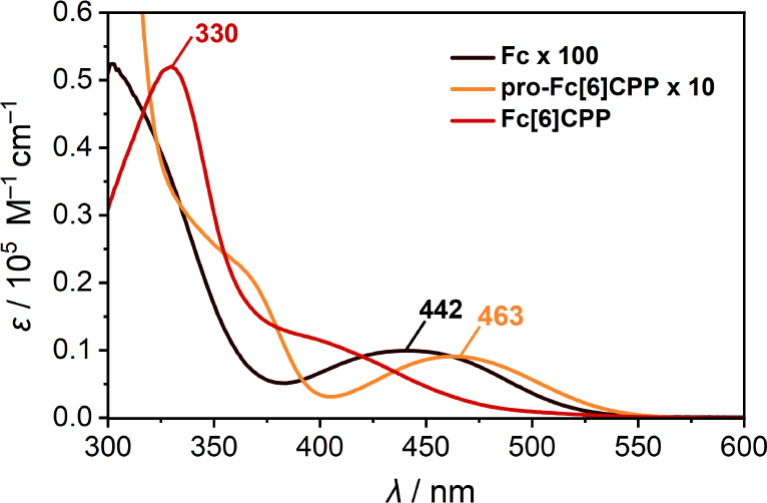
UV/vis absorption spectra of **Fc**, **pro-Fc[6]CPP**, and **Fc[6]CPP** in PhCN. For the sake of comparison, the spectra of **Fc** and **pro-Fc[6]CPP** were scaled by a factor of 100 and 10, respectively.

We examined whether embedding an Fc unit into a strained macrocyclic structure affected the optical and redox properties. The latter were determined for CH_2_Cl_2_ solutions of **Fc[6]CPP** with 0.1 M [*n*-Bu_4_N][PF_6_] as a supporting electrolyte using cyclic voltammetry (CV) and differential pulse voltammetry (DPV). A single anodic wave could be observed for **Fc[6]CPP** (Figures S7 and S8) with only a minor shift in the half-wave oxidation potential by ca. −50 mV with respect to the Fc/Fc^+^ couple. The Randles–Ševčík analysis revealed full reversibility of the oxidation process at the electrode (Figure S9). We then recorded the absorption spectra of **Fc[6]CPP** and **pro-Fc[6]CPP** (Figure 3 and Table 1). Nanohoop **Fc[6]CPP** exhibits a characteristic absorption profile observed for [*n*]CPPs with an intense transition at 330 nm (ε = 5.2 × 10^4^ M^–1^ cm^–1^) and a distinct band at ∼400 nm, which corresponds to the S_0_ → S_1_ transition in CPPs. A weak solvatochromism is observed for **Fc[6]CPP** (Figure S10). Comparison to the absorption spectrum of **pro-Fc[6]CPP**, in which the conjugation in the macrocycle is interrupted by the pro-aromatic cyclohexa-2,5-dienyls, suggests that an unresolved low-energy transition (>450 nm) may exist in **Fc[6]CPP**. This is supported by TD–DFT calculations that predict that nearly degenerate Fc-centered transitions with a low oscillatory strength represent the lowest excited states in **Fc[6]CPP**. Indeed, such *d–d* transitions are clearly visible at 442 nm (ε = 90 M^–1^ cm^–1^) in **Fc**.^51,52^ Their slight bathochromic shift with the corresponding increase of ε in **Fc[6]CPP** is the consequence of extending the π-system of the Cp ligands and decreasing the overall symmetry of the chromophore, partially allowing the *d–d* transitions as shown by the natural transition orbitals analysis of the first seven transitions in **Fc[6]CPP** (see the Supporting Information). Despite the presence of the curved *para*-phenylene segment, **Fc[6]CPP** displays no luminescence. This confirms that the lowest-energy excited state in both **pro-Fc[6]CPP** and **Fc[6]CPP** is localized on the Fc moiety, which is a known luminescence quencher.

**Table 1:** Summary of Photophysical, Redox and Structural Properties

Compd.	λ_abs_ (nm)	ε^a^ (10^3^ M^–1^ cm^–1^)	*E*_1/2_ (mV)	*E*_strain_^b^ (kcal mol^–1^)	Φ^c^ (%)
**Fc**	442	0. 1	0.0	0.0	(1.5 ± 0.3) × 10^–3^
**pro-Fc[6]CPP**	463	0. 9	-	13.7	(4.9 ± 0.4) × 10^–3^
**Fc[6]CPP**	330	52, 2.3^d^	–42^e^, –57^f^	82.6	6.0 ± 0.5

a At maximum λ_abs_.

b At D3-B3LYP/6–31++g(d)/LanL2DZ(Fe) level of theory; see the Supporting Information for the computational details.

c Absolute quantum yield of [Fe(phen)_3_]^2+^ formation in PhCN with 500 equiv of phen.

d At λ_abs_ = 472 nm.

e Determined by CV.

f Determined by DPV.

**Figure 4:**
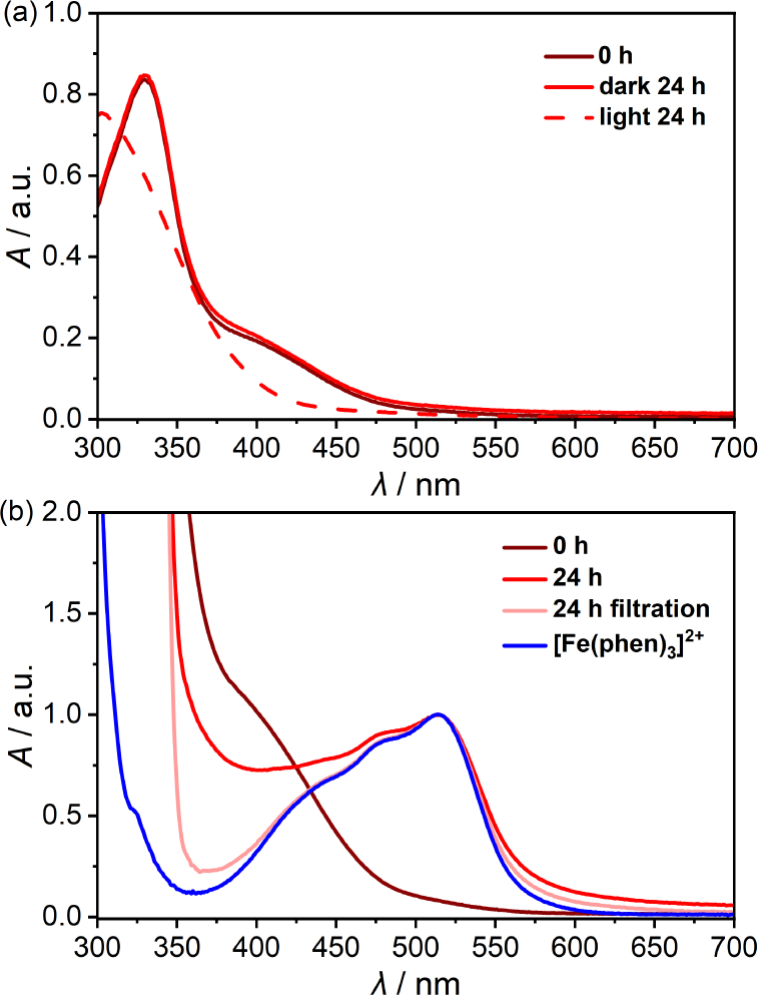
(a) **Fc[6]CPP** in PhCN (*c* ≈ 20 μM) stirred in dark (solid line) or in ambient light (dashed line) and (b) normalized absorption spectra of **Fc[6]CPP** in PhCN (*c* ≈ 60 μM, phen: 500 equiv) stirred in ambient light for 24 h (red: unfiltered, light red: filtered) and independently prepared [Fe(phen)_3_]^2+^ (blue). The spectrum at 0 h (dark red) was scaled to match the ε of [Fe(phen)_3_]^2+^.

Interestingly, while the absorption spectrum of **Fc[6]CPP** in toluene (*c* ≈ 20 μM) did not change upon exposure to ambient light (96 h, Figure S11), an equally concentrated sample of **Fc[6]CPP** in polar PhCN displayed a notable change in color within dozens of minutes. The photolysis rate is rapid when green light (λ_LED_ = 525 ± 18 nm, >10 mW) is used as the light source and the characteristic CPP band at 330 nm is no longer discernible after 30 min of irradiation (Figure S12). Note that **Fc[6]CPP** remains stable in the dark for >24 h at room temperature (Figure 4a). Similar experiments with samples of **pro-Fc[6]CPP** or **Fc** revealed a striking difference among the compounds. Both **pro-Fc[6]CPP** and **Fc** in PhCN are stable over days when exposed to daylight (Figures S13 and S14). Based on the known cases of Fe–Cp bond dissociation triggered by irradiation^16,23−27^ or mechanical force,^28–30^ we hypothesized that **Fc[6]CPP** might undergo a macrocyclic ring-opening, eventually releasing the Fe^2+^ ion and the substituted *p*-sexiphenyl (Figure 1b). To test this hypothesis, the solution of **Fc[6]CPP** was exposed to ambient light in the presence of 500 equiv of 1,10-phenanthroline (phen), which is known to bind Fe^2+^ ion to form ferroin, [Fe(phen)_3_]^2+^ complex with a distinct absorption spectrum. After 24 h, the solution was filtered, and its absorption spectrum matched that of the independently prepared sample of [Fe(phen)_3_]^2+^ (Figure 4b). The nature of the complex was also confirmed by mass spectrometry (Figure S15). Besides the photoinduced release and subsequent catch of Fe^2+^ from the nanohoop, we observed the formation of a precipitate that likely corresponds to the released *p*-sexiphenyl derivative. However, we have not yet been able to unequivocally identify the structure of the latter by mass spectrometry. We determined the yield of [Fe(phen)_3_]^2+^ formation by absorption spectroscopy (see Supporting Information) to be (80 ± 3.0) and (67 ± 5.2) % before and after filtration of the photolyzed solution, respectively. The difference stems from the presence of the precipitate, which leads to slight scattering at the absorption band of [Fe(phen)_3_]^2+^. The control experiments conducted at room temperature in the dark with a large excess of phen (500 equiv) showed minor [Fe(phen)_3_]^2+^ formation (<10% after 24 h, unfiltered, Figure S17). However, increasing the temperature to 70 °C resulted in a markedly faster transformation (Figure S18), although the reaction required a few days to reach full conversion. These observations strongly support our assumption that the strain perturbing the Fc structure markedly affects the reactivity of Fc in **Fc[6]CPP**.

**Figure 5:**
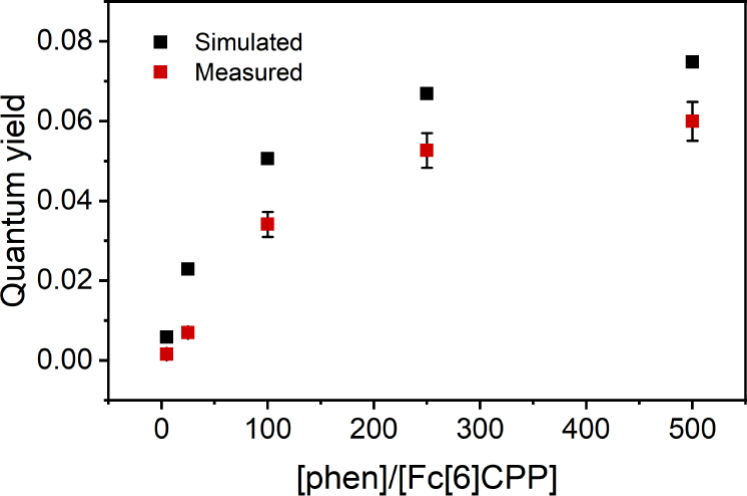
Measured (red, with standard deviation) and simulated (black, see the Supporting Information for details) quantum yields of [Fe(phen)_3_]^2+^ formation from **Fc[6]CPP** in PhCN upon irradiation with a 472 nm LED as a function of phen concentration.

**Scheme 2:**
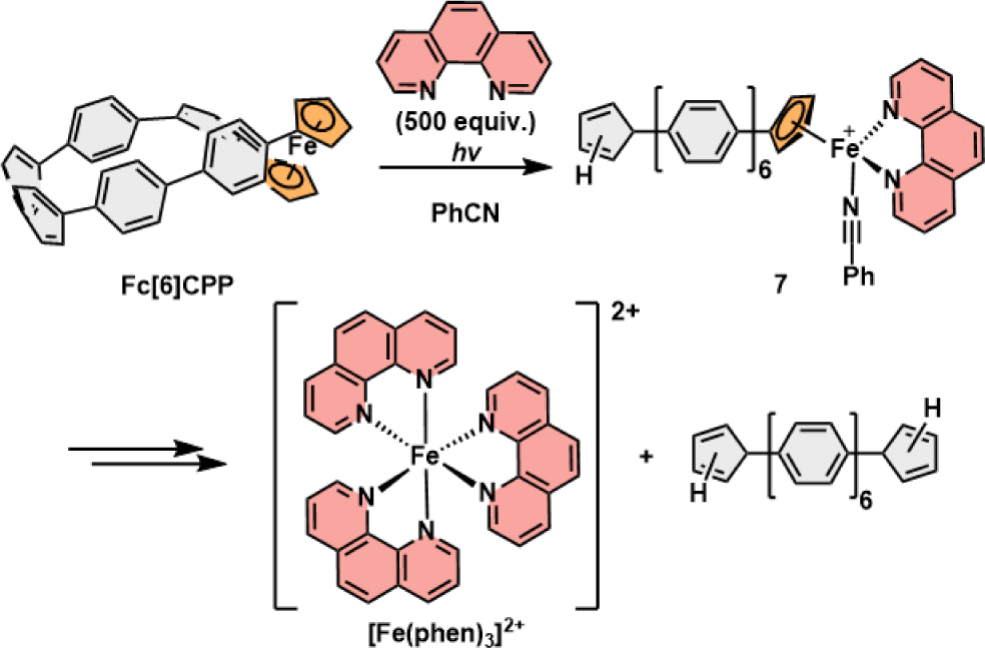
Proposed Mechanism for Dissociation in the Presence of Phen of **Fc[6]CPP** upon Irradiation

To quantify the reactivity of **Fc[6]CPP**, we compared its photochemical behavior with the less strained precursor **pro-Fc[6]CPP** as well as the unstrained **Fc** itself. For this purpose, we measured the absolute quantum yields of [Fe(phen)_3_]^2+^ formation in PhCN for all three compounds (Φ, Table 1) under identical conditions (*c* = 40 μM, 500 equiv of phen, λ_LED_ = 472 nm). The large excess of phen was necessary to detect appreciable amounts of [Fe(phen)_3_]^2+^ formed from both **pro-Fc[6]CPP** and **Fc** within hours at the full intensity of our LED source (130 mW), while **Fc[6]CPP** could be converted in seconds. Comparison of the measured Φs reveals the striking enhancement of the reactivity of **Fc[6]CPP**. While the determined Φs for **Fc** and **pro-Fc[6]CPP** are (1.5 ± 0.3) × 10^–5^ and (4.9 ± 0.4) × 10^–5^, respectively, the ring-opening in **Fc[6]CPP** is >1000-fold more efficient. The value of Φ reflects the Fc tilt angle and the strain energy in the individual compounds (Table 1), but it also depends strongly on the concentration of the phen ligand (Figure 5). The quantum yield for **Fc[6]CPP** grew from 1.6 × 10^–3^ to 0.06 with an increasing amount of phen. The Φ levels off at a large excess of phen and reaches a limiting value that compares favorably to the intersystem crossing quantum yield reported for **Fc** (Φ_ISC_ = 0.085).^53^ It suggests that the Fe–Cp bond dissociation occurs by intercepting the excited Fc unit by a ligand molecule. This step may take place efficiently only in ^**3**^**Fc** that possesses a sufficiently long excited state lifetime (τ (^**3**^**Fc**) = 90 ns vs τ (^**1**^**Fc**) = 10 ps).^53^ A simulation of the expected Φ of [Fe(phen)_3_]^2+^ formation via a triplet state, while accounting for the quenching of the oxygen present in the solution, matches the observed concentration dependence well (Figure 5). Comparison of the Φs determined in the presence and absence of oxygen with 10 equiv of phen in the solution showed a statistically significant difference (*t*(6) = 2.26, *p* < 0.05), confirming that the reaction occurs via the triplet state of **Fc[6]CPP**. Indeed, the DFT-calculated spin density shows an antibonding character of the Fe–Cp bond in triplet **Fc[6]CPP** and the presence of an electron hole at the Fe center (Figure S35). This increases iron electrophilicity^47,54^ promoting the bond dissociation when attacked by a nucleophilic solvent or external ligand.^16^ Such a polar transition state is in agreement with our observation that the photolysis of **Fc[6]CPP** is facile in polar PhCN (Figure 4a) and inefficient in toluene (Figure S11). It has also been reported that electron-donating solvents promote the Fe–Cp bond dissociation.^25,55−57^ We thus expect that a half-sandwich complex **7** (Scheme 2) is formed as the first reaction step with a temporarily coordinated solvent and the *p*-sexiphenyl ligand still attached to the Fe via the remaining Cp. Subsequently, excess phen must rapidly replace the labile ligands because we do not observe the formation of any intermediates by UV–vis spectroscopy with 250 ms sampling. A complex such as **7** has often been proposed^58−60^ as an intermediate in the dissociation of [1]- and [2]ferrocenophanes, but it has never been detected. Note that no free Fe^2+^ forms directly from **Fc[6]CPP**, which we verified by photolysis of the **Fc[6]CPP** before the addition of phen (500 equiv) to the PhCN solution. Here, we observed only a slow formation (>72 h) of [Fe(phen)_3_]^2+^ in the dark. The process proceeded somewhat faster under ambient light. However, complexation of Fe(OTf)_2_ in PhCN treated with excess phen occurs rapidly (<10 min, compare Figures S29–S31).

As shown above, **Fc[6]CPP** undergoes a remarkably clean ring-opening reaction in a polar environment upon irradiation with benign light. The elevated strain energy of the nanohoop makes photochemical Fe–Cp bond dissociation and subsequent Fe^2+^ release and trapping particularly efficient. Given the crucial role of iron in various biological processes, we decided to probe the photoreactivity of **Fc[6]CPP** in a mixed aqueous/organic medium due to the negligible solubility of the nanohoop in pure water. The irradiation of **Fc[6]CPP** in a mixture of H_2_O/THF (*v/v* = 1:1, 500 equiv phen) with green light resulted in the formation of [Fe(phen)_3_]^2+^. Here, [Fe(phen)_3_]^2+^ formed cleanly in (81 ± 1.1) % yield after filtration (Figure S33) with ∼75% efficiency compared to the experiment in neat PhCN. The iron release from **Fc[6]CPP** upon irradiation is thus a highly effective process also in polar protic solvents, which creates exciting opportunities for biological applications such as delivery and dosing of iron controlled by light, e.g., to study their biochemical pathways or to induce ferroptosis.^61−63^ It is important to note here that although the macrocyclic structure of **Fc[6]CPP** irreversibly opens upon irradiation without phen, the presence of phen alters the iron release mechanism and significantly enhances the overall release rate. The nature and the kinetic stability of the formed primary complex in water in the absence of phen require detailed mechanistic scrutiny to reveal how it might be decomposed to release iron using nucleophiles ubiquitous in biological systems.

The tunability of strain in carbon nanohoops by precision synthesis may represent a great tool to uncover and control the reactivity in similar types of metallamacrocycles such as the one designed in this work, which could aid the development of new redox-active or supramolecular systems that are responsive to light or mechanical force. Work along these lines is currently ongoing in our laboratories.

## Conclusions

In summary, we synthesized a highly strained π-conjugated macrocycle **Fc[6]CPP** that incorporates ferrocene. Its structure was unequivocally characterized by X-ray diffraction analysis and quantum chemical calculations. The high strain in **Fc[6]CPP** transforms the reactivity of photostable ferrocene into a photoactivatable carrier of iron that is released via iron–cyclopentadienyl bond cleavage and easily trapped with phenanthroline to form a ferroin complex. The observed clean and high-yielding reaction is 3 orders of magnitude more efficient than that for ferrocene and demonstrates the power of molecular strain to uncover and control the reactivity of an otherwise stable metallocene complex. Furthermore, we demonstrated that this system efficiently releases iron in a mixed aqueous/organic environment, suggesting that its operation can be translated to a biological context. We believe that this work will thus motivate new developments in the fields of responsive materials, photocages, or organometallic and host–guest chemistry.

## Supporting Information Available

Experimental procedures and characterization data for all new compounds; experimental and computational details (PDF)
