# Supplementary material for: Strain-Induced Photochemical Opening of Ferrocene[6]cycloparaphenylene: Uncaging of Fe2+ with Green Light
Source: J Am Chem Soc. 2025 Jan 17;147(12):10231–7. doi: 10.1021/jacs.4c15818 (PMC11951145; doi:10.1021/jacs.4c15818)
Supplement: Supplementary file 1 — ja4c15818_si_001.pdf [file ja4c15818_si_001.pdf]

Supporting Information

Strain-Induced Photochemical Opening of Ferrocene[6]cycloparaphenylene: Uncaging of Fe^{2+} with Green Light

Remigiusz B. Kręcijasz[a], Juraj Malinčík[a], Simon Mathew[a], Peter Štacko*[b], Tomáš Šolomek*[a]

[a]van 't Hoff Institute for Molecular Sciences, University of Amsterdam, PO Box 94157, 1090 GD Amsterdam, The Netherlands

[b]Department of Chemistry, University of Zurich, Wintherthurerstrasse 190, 8057 Zurich, Switzerland

E-mail: t.solomek@uva.nl; peter.stacko@chem.uzh.ch

Contents

1. General information 2

2. Experimental procedures 3

3. Single Crystal X-Ray Diffraction 6

4. Electrochemical Measurements 9

5. Photophysical studies 11

5.1 Absorption spectra 11

5.2 HRMS spectra of photolyzed solution 13

5.3 Yield of $[Fe(phen)_3]^{2+}$ complex formation 14

5.4 Control experiments w/o light 15

5.6 Quantum yields of $[Fe(phen)_3]^{2+}$ complex formation 16

5.7 **Quantum yield simulations** 21

5.8 Mechanistic insights 23

5.9 Experiments in THF/H_2O 24

6. DFT calculations 26

6.1 General remarks 26

6.2 Strain energy 26

6.3 Frontier molecular orbitals 27

6.4 Spin density plot 28

6.5 Time dependent DFT calculations 28

7. ^1H and ^{13}NMR spectra 33

8. Mass spectra 40

9. References 41

1. General information

All commercially available reagents and chemicals were purchased from Sigma Aldrich, TCI, Fluorochem and Fisher Scientific and used as received without further purification, unless stated otherwise. Anhydrous solvents were purchased from Acros and stored under molecular sieves (4 Å). Technical solvents were bought from VWR International and Biosolve, and were used as received. Unless otherwise stated, all glassware used to perform moisture-sensitive reactions was oven-dried at 120 °C overnight, assembled hot and allowed to cool down to room temperature under a stream of argon, or flame-dried under high vacuum and filled with argon. All reactions that require heating were conducted in an oil bath and the indicated temperature corresponds to the temperature of the oil bath. Flash column chromatography was performed using silica gel 60 Å (230–400 mesh particle size) from Supelco®. Thin layer chromatography (TLC) was performed on silica gel plates F_{254} 60 (aluminum supported) from Supelco® using UV (254 nm) visualization. Recycling gel permeation chromatography (GPC) was performed on LaboAce 5060 preparative HPLC from Japan Analytical Services using Chloroform as the solvent. ^{1}H and ^{13}C NMR spectra were recorded on a Bruker Avance II 400 or a Bruker Avance III HD 400 spectrometer (^{1}H: 400 MHz, ^{13}C: 101 MHz), or Bruker Avance III HD 300 (^{1}H: 300 MHz, ^{13}C: 75 MHz). Chemical shifts (δ) were reported in parts per million (ppm) referenced to residual solvent peak (^{1}H: 7.26 ppm for $CDCl_3$, 5.32 ppm for CD_2Cl_2; ^{13}C: 77.16 ppm for $CDCl_3$, 53.84 ppm for CD_2Cl_2). Multiplicities are given as s (singlet), d (doublet), t (triplet), q (quadruplet), m (multiplet), and br (broad). Coupling constants (*J*) are reported in hertz (Hz). High resolution mass spectra (HRMS) were recorded on ThermoScientific LTQ Orbitrap XL mass instrument using nanoelectrospray (NSI-MS) or electron ionization (EI–MS) or on an AccuTOF LC, JMS-T100LP mass spectrometer (JEOL, Japan). UV–Vis spectra were recorded with a double beam spectrophotometer Shimadzu UV2700 equipped with a deuterium lamp (190–350 nm), a halogen lamp (330–900 nm) and a photomultiplier (Hamamatsu R928). Single–crystal X-ray diffraction data measured on a Bruker D8 Quest Eco diffractometer using graphite-monochromated (Triumph) Mo Kα radiation (λ = 0.71073 Å) and a CPAD Photon III C14 detector. The sample was cooled with N_2 to 100 K with a Cryostream 700 (Oxford Cryosystems).

2. Experimental procedures

1,1′-diiodoferrocene[1] and **1**[2] were prepared according to reported protocols.

Preparation of compound **2**

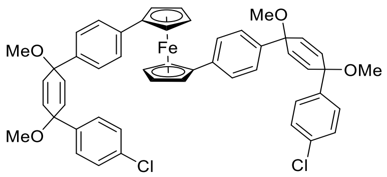

Compound **1** (3.10 g, 6.85 mmol, 3 equiv.) and NaOH (365 mg, 9.14 mmol, 4 equiv., CAS: 1310-73-2) were introduced in a Schlenk flask. The flask was evacuated and refilled with argon three times. Then, 1,1′-diiodoferrocene (1.00 g, 2.28 mmol, 1 equiv.) was added, followed by DME (30 mL) and water (6 mL). The mixture was purged with argon for 15 min, after which Pd(dppf)Cl_2 (84 mg, 0.114 mmol, 0.05 equiv., CAS: 72287-26-4) was added. The reaction mixture was stirred at 85 °C for 20 h, then allowed to cool down to room temperature, diluted with EtOAc (20 mL) and washed once with water (25 mL). The aqueous phase was extracted with EtOAc (3 × 15 mL). The combined organic layers were washed with brine, dried over Na_2SO_4 and concentrated in vacuo to afford a dark thick oil. The crude product was dissolved in a minimal amount of CH_2Cl_2 and an excess of MeOH was slowly added without disturbing the CH_2Cl_2 phase. The next day, the solution was filtered to give the pure product as the precipitate. Because the filtrate still contained some product, the purification with CH_2Cl_2/MeOH was repeated two more times. The repeated precipitations provided **2** as a brown solid (993 mg, 1.19 mmol, 52 %).

Brown solid; R_f = 0.42 (SiO_2, EtOAc: *n*-pentane = 1:4).

^{1}H NMR (300 MHz, CD_2Cl_2): δ = 7.45 – 7.10 (m, 16H), 6.14 (d, *J* = 10.3 Hz, 4H), 6.04 (d, *J* = 10.3 Hz, 4H), 4.44 (t, *J* = 1.9 Hz, 4H), 4.18 (t, *J* = 1.9 Hz, 4H), 3.42 (s, 12H).

^{13}C NMR (75 MHz, CD_2Cl_2): δ = 142.9, 141.4, 138.2, 134.3, 133.5, 133.3, 128.7, 128.0, 126.3, 126.3, 85.9, 75.0, 74.8, 71.3, 68.4, 52.2, 52.2.

HRMS (ESI+): *m/z* $[M]^+$ calcd for $C_{50}H_{44}Cl_2O_4Fe$: 834.1968; found: 834.1979.

Preparation of compound **3**

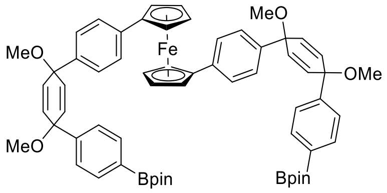

Compound **2** (779 mg, 0.932 mmol, 1 equiv.), bis(pinacolato)diboron (947 mg, 3.73 mmol, 4 equiv., CAS: 73183-34-3), $Pd_2(dba)_3$ (43 mg, 0.047 mmol, 0.05 equiv., CAS: 51364-51-3), XPhos (89 mg, 0.186 mmol, 0.2 equiv., CAS: 564483-18-7) and dry potassium acetate (732 mg, 7.46 mmol, 8 equiv., CAS:

127-08-2) were introduced to a Schlenk flask and purged with argon for 15 min. Anhydrous 1,4-dioxane (20 mL) purged with argon for 15 min was then added via cannula. The reaction mixture was stirred at 110 °C for 16 h. After the addition of water (25 mL), the reaction was extracted with CH_2Cl_2 (3 × 25 mL). The combined organic layers were washed with brine, dried over Na_2SO_4 and concentrated in vacuo to afford a brown solid. Crude product was washed several times with *n*-hexanes to afford **3** as brown solid (769 mg, 0.755 mmol, 81 %).

Brown solid; R_f = 0.33 (SiO_2, EtOAc: *n*-pentane = 1:4).

^{1}H NMR (300 MHz, $CDCl_3$): δ = 7.78 (d, *J* = 7.8 Hz, 4H), 7.44 (d, *J* = 7.8 Hz, 4H), 7.35 – 7.20 (m, 8H), 6.23 – 6.00 (m, 8H), 4.48 (t, *J* = 1.9 Hz, 4H), 4.15 (t, *J* = 1.9 Hz, 4H), 3.45 (s, 12H), 1.34 (s, 24H).

^{13}C NMR (75 MHz, $CDCl_3$): δ = 146.7, 141.0, 138.1, 135.0, 133.6, 133.2, 126.2, 126.1, 125.5, 85.7, 83.9, 75.1, 74.9, 71.2, 68.0, 52.1, 52.1, 25.0, C-B signal not observed.

HRMS (NSI): *m/z* $[M]^+$ calcd for $C_{62}H_{68}O_8B_2Fe$: 1018.4444; found: 1018.4418.

Preparation of **pro-Fc[6]CPP**

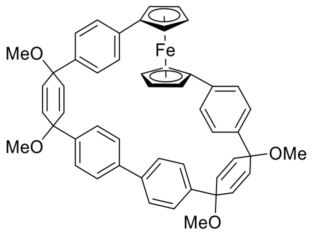

We modified a literature procedure:[3] To the solution of compound **3** (299 mg, 0.294 mmol, 1 equiv.) in THF (300 mL) were added Pd(dppf)Cl_2 (20 mg, 0.03 mmol, 0.1 equiv., CAS: 72287-26-4) and boric acid (91 mg, 1.470 mmol, 5 equiv., 10043-35-3). The reaction mixture was heated to 40 °C and meanwhile it was purged with oxygen for 10 min. Potassium fluoride (17 mg, 0.294 mmol, 1 equiv., CAS: 7789-23-3) in water (30 mL) was added. The reaction was stirred at 40 °C open to air for 20 h. The THF was removed in vacuo and the resulting solution was extracted with CH_2Cl_2 (3 × 20 mL). The combined organic layers were washed with water (2 × 25 mL), dried over Na_2SO_4 and concentrated in vacuo to afford a brown solid. Crude product was purified by flash chromatography (SiO_2, EtOAc: *n*-pentane = 1:9 → EtOAc: *n*-pentane = 3:7) to afford **pro-Fc[6]CPP** as orange solid (125 mg, 0.163 mmol, 56 %). The impure fractions were further purified by GPC to afford the pure macrocycle (17 mg, 0.022 mmol, 7%, total yield = 63%).

Orange solid; R_f = 0.26 (SiO_2, EtOAc: *n*-pentane = 1:4).

^{1}H NMR (400 MHz, $CDCl_3$): δ = 7.51 (d, *J* = 8.5 Hz, 4H), 7.37 (d, *J* = 8.5 Hz, 4H), 7.28 (d, *J* = 8.5 Hz, 4H), 7.01 (d, *J* = 8.5 Hz, 4H), 6.33 – 6.28 (m, 4H), 6.21 – 6.18 (m, 2H), 6.12 – 6.09 (m, 2H), 4.37 (s, 2H), 4.29 (s, 2H), 4.03 (s, 2H), 3.98 (s, 2H), 3.41 (s, 6H), 3.36 (s, 6H).

^{13}C NMR (101 MHz, $CDCl_3$): 141.3, 140.4, 139.7, 138.2, 133.8, 133.6, 133.6, 133.3, 127.4, 127.1, 126.8, 125.6, 86.7, 76.0, 75.9, 71.0, 70.9, 70.5, 64.9, 52.4, 52.1.

HRMS (NSI): *m/z* $[M]^+$ calcd for $C_{50}H_{44}O_4Fe$: 764.2584; found: 764.2601.

Preparation of compound **Fc[6]CPP**

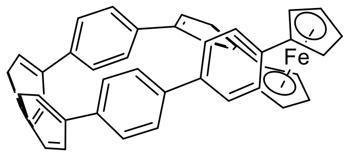

A 0.08 M H_2SnCl_4 solution was prepared by stirring $SnCl_2{\cdot}2H_2O$ (90.2 mg, 0.4 mmol, CAS: 10025-69-1) and concentrated HCl (12 M) (67 µL, 0.8 mmol, CAS: 7647-01-0) in THF (5 mL) at room temperature for 30 min. After this time, H_2SnCl_4 solution (0.08 M, 3.5 mL, 0.282 mmol, 3.6 equiv.) was added to **pro-Fc[6]CPP** (60.0 mg, 0.079 mmol, 1 equiv.) and the reaction was stirred at room temperature for 2 h. The reaction was quenched with 1 M NaOH (2 mL) and the aqueous layer was extracted with CH_2Cl_2 (3 × 10 mL). The organic layers were washed with brine, dried over Na_2SO_4 and concentrated in vacuo to afford **Fc[6]CPP** as dark red solid (38.4 mg, 0.060 mmol, 76%).

Dark red solid; R_f = 0.38 (SiO_2, EtOAc: *n*-pentane = 1:4).

^{1}H NMR (300 MHz, $CDCl_3$): δ = δ 7.50 – 7.43 (m, 12), 7.40 (d, *J* = 8.8 Hz, 4H), 7.24 (d, *J* = 8.5 Hz, 4H), 7.18 (d, *J* = 8.4 Hz, 4H), 4.37 (t, *J* = 1.8 Hz, 4H), 4.20 (t, *J* = 1.9 Hz, 4H).

^{13}C NMR (75 MHz, $CDCl_3$): δ = 138.8, 138.7, 138.3, 137.8, 137.4, 136.8, 128.7, 128.3, 127.9, 127.9, 127.7, 127.3, 90.4, 69.9, 68.0.

HRMS (NSI): *m/z* $[M]^+$ calcd for $C_{46}H_{32}Fe$: 640.1848; found: 640.1868.

3. Single Crystal X-Ray Diffraction

X-ray diffraction data of **pro-Fc[6]CPP** and **Fc[6]CPP** were measured on a Bruker D8 Quest Eco diffractometer using graphite-monochromated (Triumph) Mo Kα radiation (λ = 0.71073 Å) and a CPAD Photon III C14 detector. The sample was cooled with N_2 to 100 K with a Cryostream 700 (Oxford Cryosystems). Intensity data were integrated using the SAINT software.[4] Absorption correction and scaling was executed with SADABS.[5] The structures were solved using intrinsic phasing with the program SHELXT 2018/2[6] against F2 of all reflections.

The crystal structure contained two voids (total solvent accessible volume = 144 Å^3), containing THF solvent within the asymmetric unit could not be refined reliably. Thus, the SQUEEZE[7] procedure in PLATON[8] (version 141123) was applied, accounting for 38 electrons per unit cell, congruent with the presence of 1 THF molecule (40 e^-/molecule) in the unit cell (Z=2).

Least-squares refinement was performed with SHELXL-2018/3.[9] All non-hydrogen atoms were refined with anisotropic displacement parameters. The hydrogen atoms were introduced at calculated positions with a riding model. CheckCIF revealed no A- or B-level alerts. The X-ray crystallographic data for **pro-Fc[6]CPP** (2373507) and **Fc[6]CPP** (2373508) was deposited at the Cambridge Crystallographic Data Centre (CCDC).

Table S1. Crystallographic data and structure refinement details for **pro-Fc[6]CPP** and **Fc[6]CPP**.

**CCDC number**	**2373507**	**2373508**
**Empirical formula**	$C_{54.56}H_{50}FeO_4$	$C_{48}H_{36}FeO_{0.50}$
**Formula weight**	825.51	676.62
**Temperature [K]**	100(2)	100(2)
**Crystal system**	triclinic	monoclinic
**Space group (number)**	$P\bar{1}$ (2)	$P2_1$ (4)
a **[Å]**	12.1840(9)	11.5096(6)
b **[Å]**	20.5519(16)	10.3463(5)
c **[Å]**	27.401(2)	14.0369(7)
**α [°]**	75.872(3)	90
**β [°]**	85.312(3)	95.431(2)
**γ [°]**	73.239(3)	90
**Volume [Å^3]**	6370.7(8)	1664.04(14)
Z	6	2
**ρ_{calc} [gcm^{-3}]**	1.291	1.350
**μ [mm^{-1}]**	0.403	0.490
***F*(000)**	2612	708
**Crystal size [mm^3]**	0.417×0.320×0.145	0.303×0.189×0.166
**Crystal colour**	orange	orange
**Crystal shape**	block	block
**Radiation**	MoK_α (λ=0.71073 Å)	MoK_α (λ=0.71073 Å)
**2θ range [°]**	3.83 to 66.34 (0.65 Å)	5.30 to 55.11 (0.77 Å)
**Index ranges**	−18 ≤ h ≤ 18<br>−31 ≤ k ≤ 31<br>−42 ≤ l ≤ 42	−14 ≤ h ≤ 14<br>−13 ≤ k ≤ 13<br>−18 ≤ l ≤ 18
**Reflections collected**	666505	45540
**Independent reflections**	48544, R_{int} = 0.0583, R_{sigma} = 0.0235	7574, R_{int} = 0.0376, R_{sigma} = 0.0282
**Completeness to θ = 25.242°**	99.8 %	99.7 %
**Data / Restraints / Parameters**	48544/130/1659	7574/7/462
**Goodness-of-fit on F^2**	1.056	1.045
**Final *R* indexes [I≥2σ(I)]**	R_1 = 0.0432, wR_2 = 0.1116	R_1 = 0.0350, wR_2 = 0.0880
**Final *R* indexes [all data]**	R_1 = 0.0548, wR_2 = 0.1207	R_1 = 0.0387, wR_2 = 0.0913
**Largest peak/hole [eÅ^{-3}]**	1.26/-1.12	1.18/-0.30
**Flack X parameter**		0.079(17)

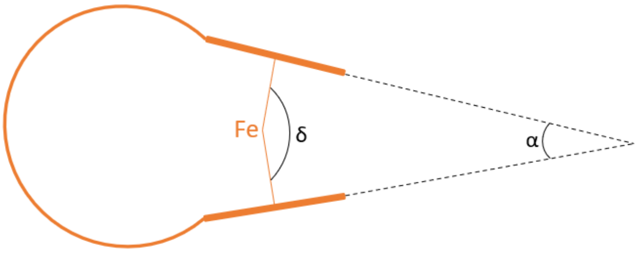

Figure S1. Definition of angles α and δ used to characterize structural distortion in ferrocenophanes.

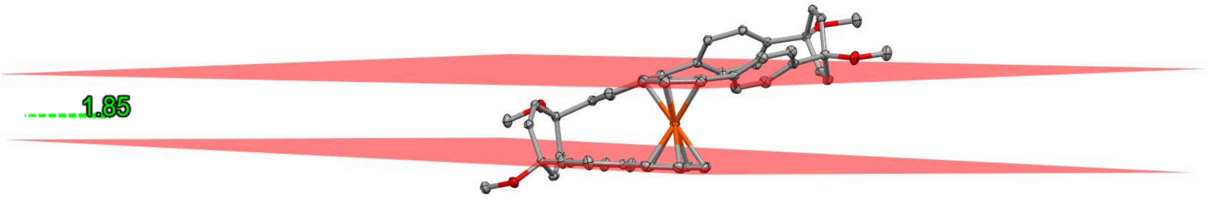

Figure S2. Tilt angle α between Cp rings in **pro-Fc[6]CPP** (conformer 2, enantiomer 1) (ellipsoids shown at 50% probability; all hydrogen atoms and solvent were omitted for clarity).

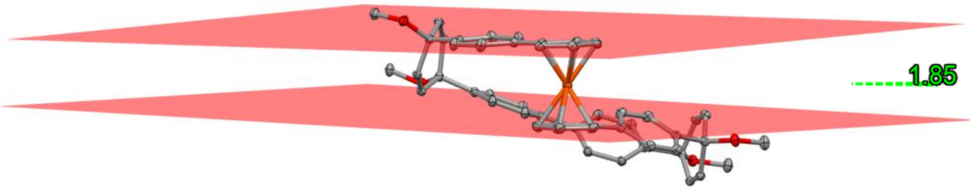

Figure S3. Tilt angle α between Cp rings in **pro-Fc[6]CPP** (conformer 2, enantiomer 2) (thermal ellipsoids shown at 50% probability; all hydrogen atoms and solvent were omitted for clarity).

Table S2. Angles α and δ in different conformers of **pro-Fc[6]CPP**.

	α (°)	δ (°)
**Conformer 1**	0.67	179.75
**Conformer 2**	1.85	179.09
**Conformer 3**	4.59	176.81

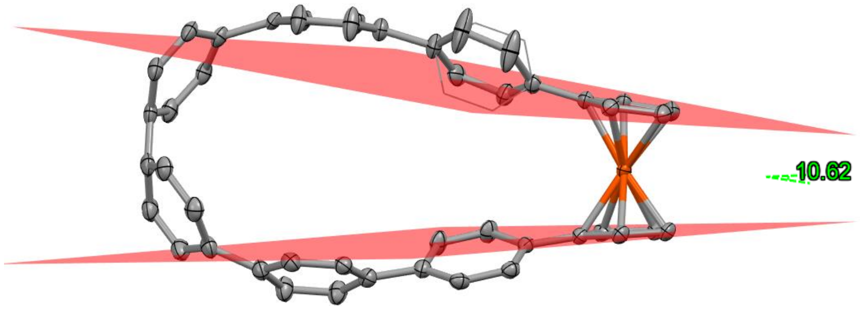

Figure S4. Tilt angle α between Cp rings in **Fc[6]CPP** (thermal ellipsoids shown at 50% probability; all hydrogen atoms and solvent were omitted for clarity).

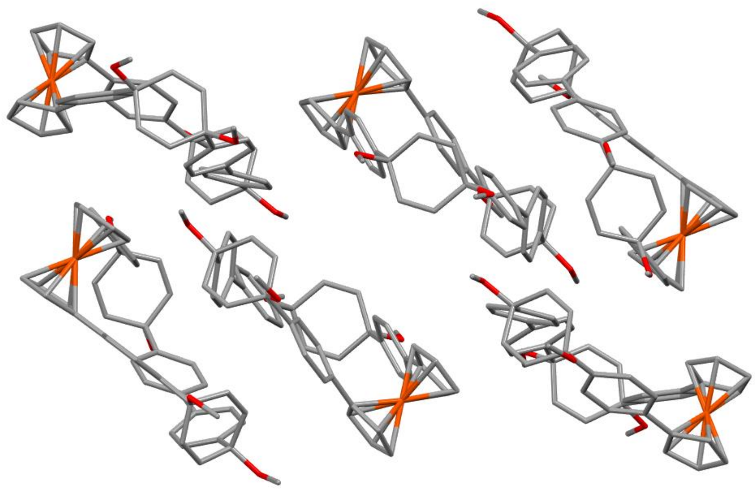

Figure S5. Packing of **pro-Fc[6]CPP** (all hydrogen atoms and solvent were omitted for clarity).

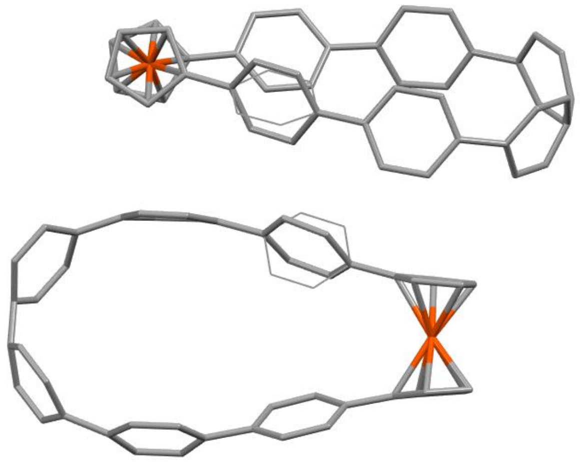

Figure S6. Packing of **Fc[6]CPP** (all hydrogen atoms and solvent were omitted for clarity).

4. Electrochemical Measurements

Cyclic voltammetry (CV) and differential pulse voltammetry (DPV) experiments were conducted using a Metrohm Autolab potentiostat running Nova 1.11 software. A glassy carbon working electrode (diameter of electrode disk 2 mm ±0.1 mm), a platinum counter electrode and a leakless Ag/AgCl reference electrode were used. Measurements of CH_2Cl_2 solutions of a sample (1 mM) were conducted at room temperature under a N_2 atmosphere in the dark in the presence of a supporting electrolyte (*n*-Bu_4NPF_6, 0.1 M). Degassed (3 × freeze-pump-thaw cycle) CH_2Cl_2 was used. Supporting electrolyte *n*-Bu_4NPF_6 was recrystallized from ethanol three times. After the measurement, ferrocene (Fc) was added to the sample solution and the potentials were calibrated with respect to the ferrocene/ferrocenium couple (Fc/Fc^+) at 0.64 V[10] vs Ag/AgCl.

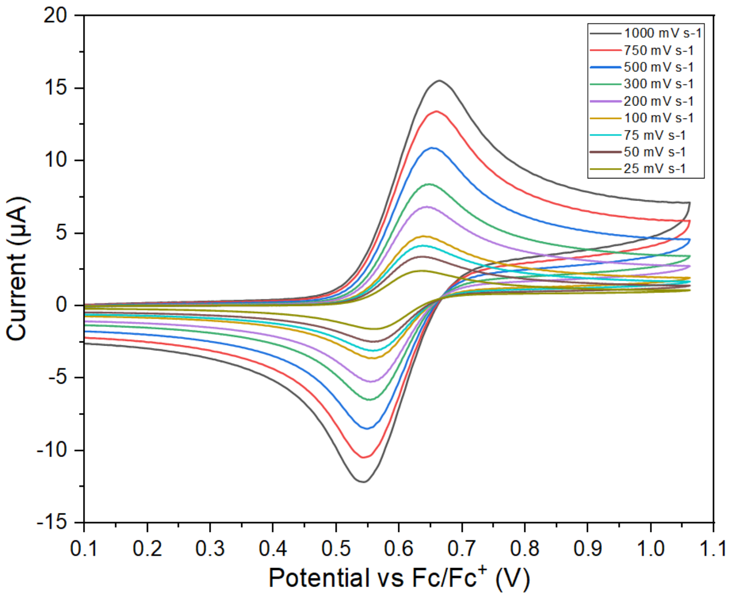

Figure S7. Cyclic voltammetry of **Fc[6]CPP** recorded in 0.1 M *n*-Bu_4NPF_6/CH_2Cl_2 at different scan rates (referenced against Fc/Fc^+).

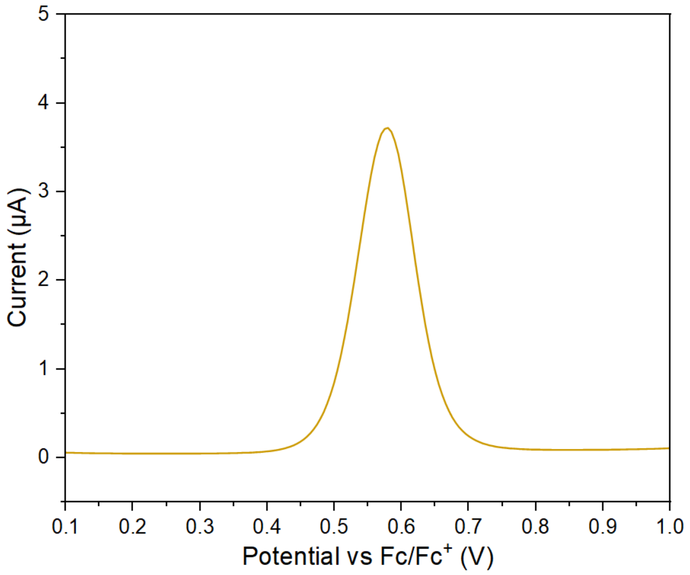

Figure S8. Differential pulse voltammetry of **Fc[6]CPP** recorded in 0.1 M *n*-Bu_4NPF_6/CH_2Cl_2 at a scan rate of 100 mV s^{-1} (referenced against Fc/Fc^+).

Table S3. $E_{1/2}$ values of **Fc[6]CPP** obtained by CV and DPV and referenced against Fc/Fc$^+$ (three independent samples).

	$E_{1/2}$ vs Fc/Fc$^+$ (V) (from CV at v = 100 mV s^{-1})	$E_{1/2}$ vs Fc/Fc$^+$ (V) (from DPV at v = 100 mV s^{-1})
**Measurement 1**	0.591	0.580
**Measurement 2**	0.606	0.590
**Measurement 3**	0.600	0.580
**Average**	$0.599 \pm 6.01 \cdot 10^{-3}$	$0.583 \pm 4.75 \cdot 10^{-3}$

The reversibility was assessed by plotting the square root of the scan rate (v) against the anodic peak current ($i_{p,a}$) from CV (Randles–Ševčík equation).

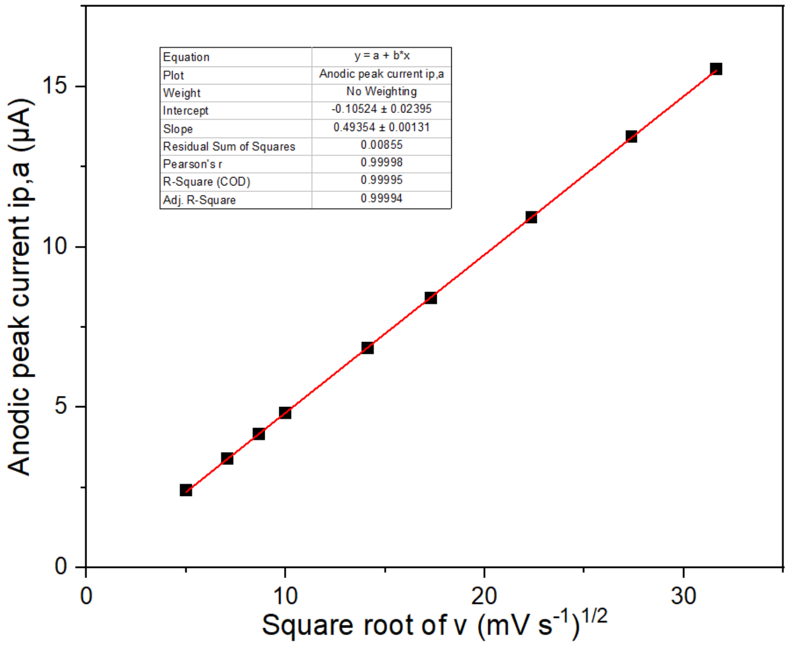

Figure S9. Square root of the scan rate (v) against the anodic peak current ($i_{p,a}$) showing the reversibility of **Fc[6]CPP** oxidation.

5. Photophysical studies

5.1 Absorption spectra

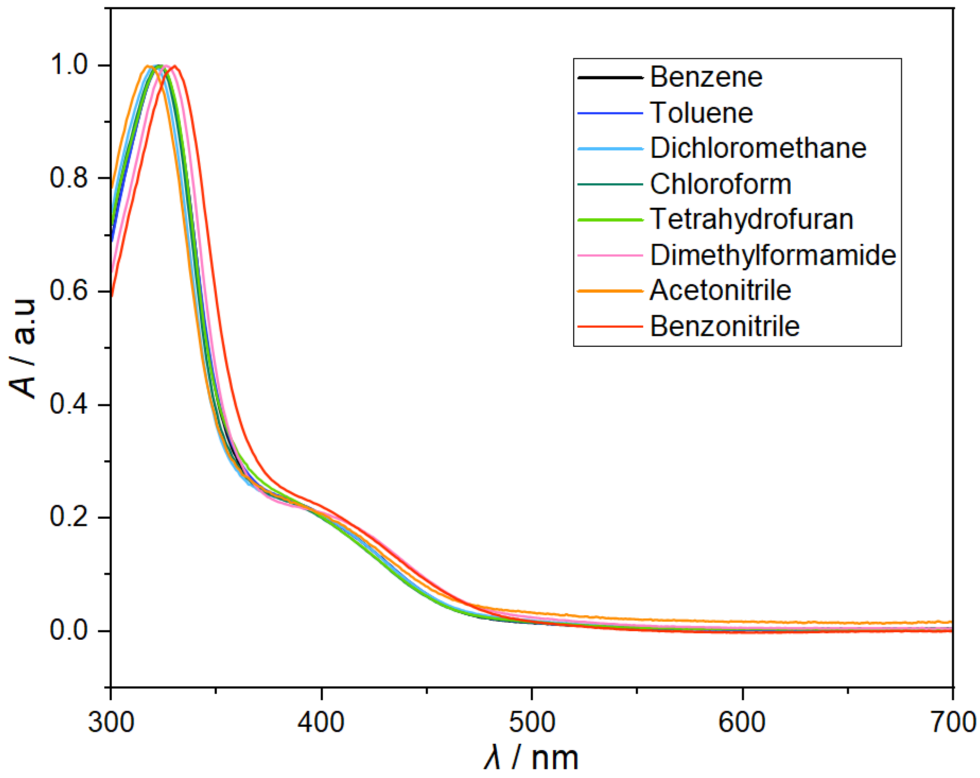

Figure S10. Normalized absorption spectra of **Fc[6]CPP** (≈ 20 µM) in different solvents.

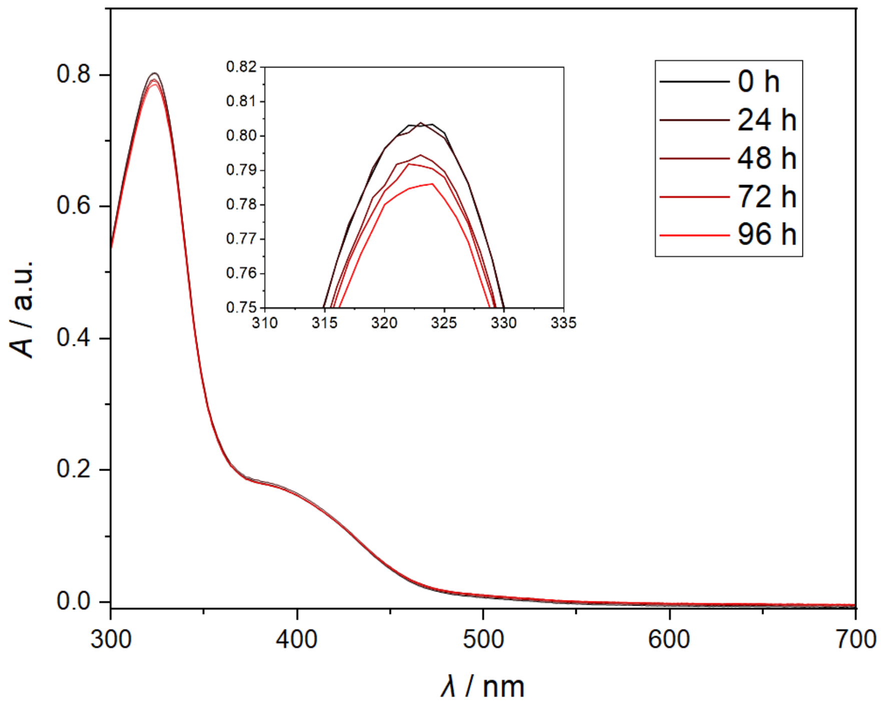

Figure S11. Absorption spectra of **Fc[6]CPP** in toluene (≈ 20 µM) exposed to daylight over 96 h. Inset shows the zoomed maxima.

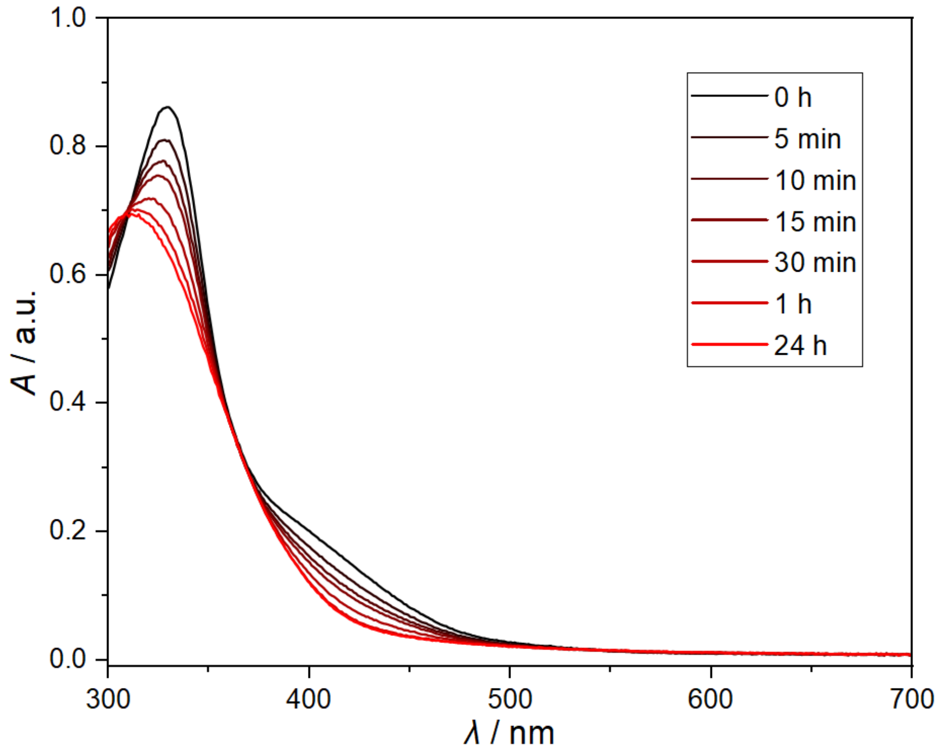

Figure S12. Absorption spectra of **Fc[6]CPP** in PhCN (≈ 20 µM) irradiated with 525 nm light.

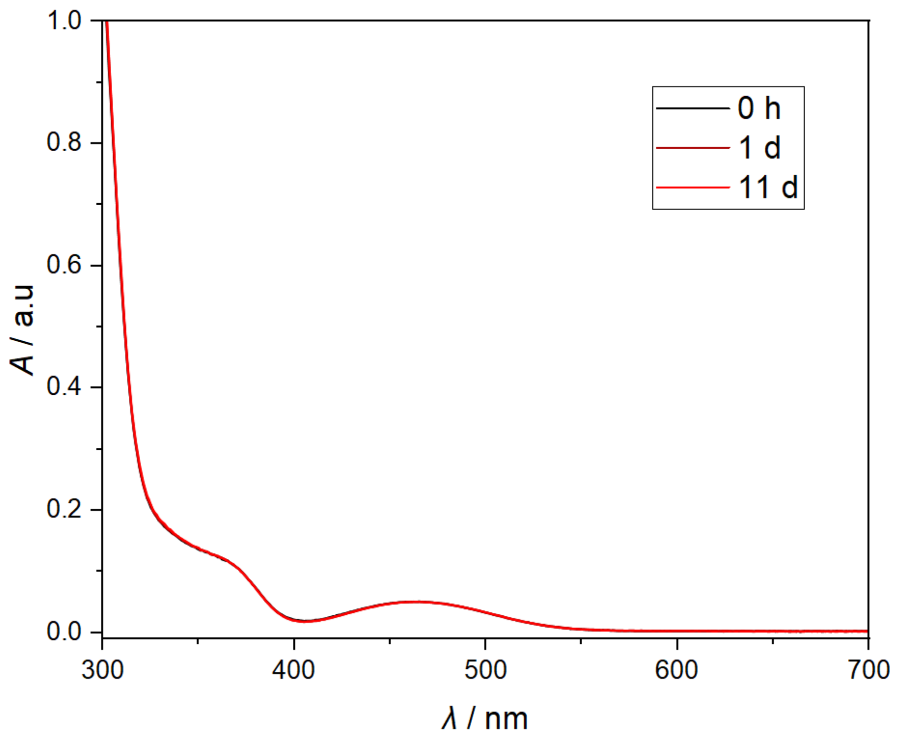

Figure S13. Absorption spectra of **pro-Fc[6]CPP** in PhCN (≈ 40 µM) exposed to daylight over 11 days.

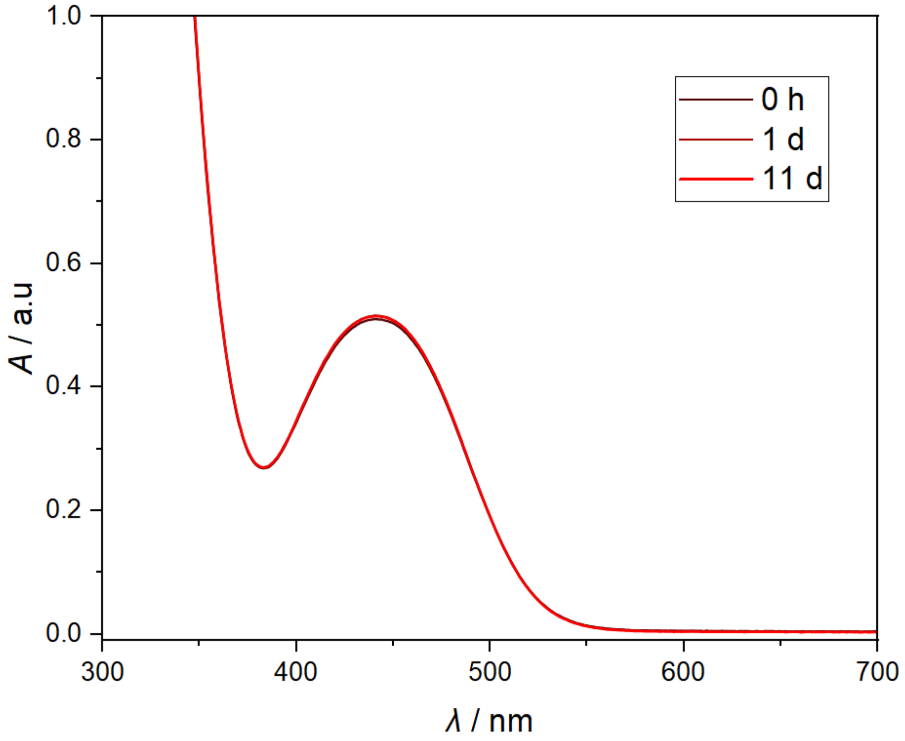

Figure S14. Absorption spectra of **Fc** in PhCN (≈ 6.5 mM) exposed to daylight over 11 days.

5.2 HRMS spectra of photolyzed solution

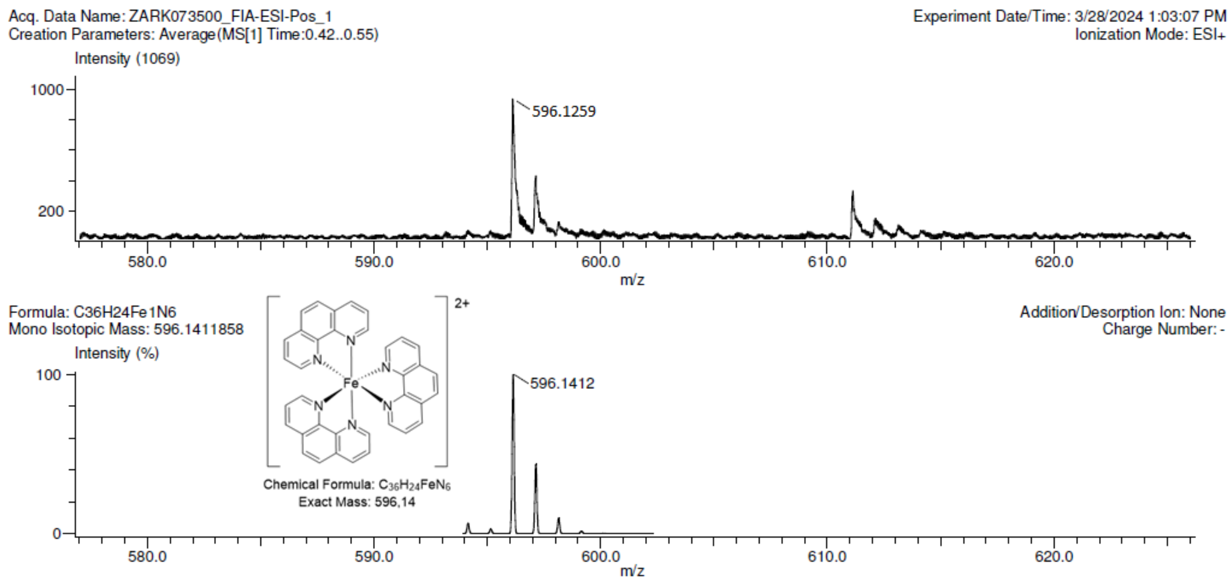

Figure S15. HRMS spectrum of $Fe(phen)_3]^{2+}$ complex obtained after stirring a **Fc[6]CPP** solution in PhCN (≈ 60 µM) with phen (500 equiv.) in ambient light at room temperature for 24 h (top: obtained spectrum, bottom: simulated spectrum).

5.3 Yield of $[Fe(phen)_3]^{2+}$ complex formation

- Molar extinction coefficient ε of $[Fe(phen)_3]^{2+}$ complex

The $[Fe(phen)_3]^{2+}$ complex solution was prepared by reacting $Fe(OTf)_2$ (20.00 mg, 0.057 mmol, 1 equiv.) with 1,10-phenanthroline (phen) (50.91 mg, 0.283 mmol, 5 equiv.) in 10.00 mL of PhCN (c = 0.00565 M) over 24 h at room temperature. This solution was used to prepare five samples with concentrations between 20-100 µM which were used to measure absorption spectra. The obtained value ε (513 nm) = 11,171 L mol^{-1} cm^{-1} is similar to values from literature: ε (510 nm) = 11,500 L mol^{-1} cm^{-1} in water[11] and ε (510 nm) = 11,100 L mol^{-1} cm^{-1} (solvent not indicated).[12]

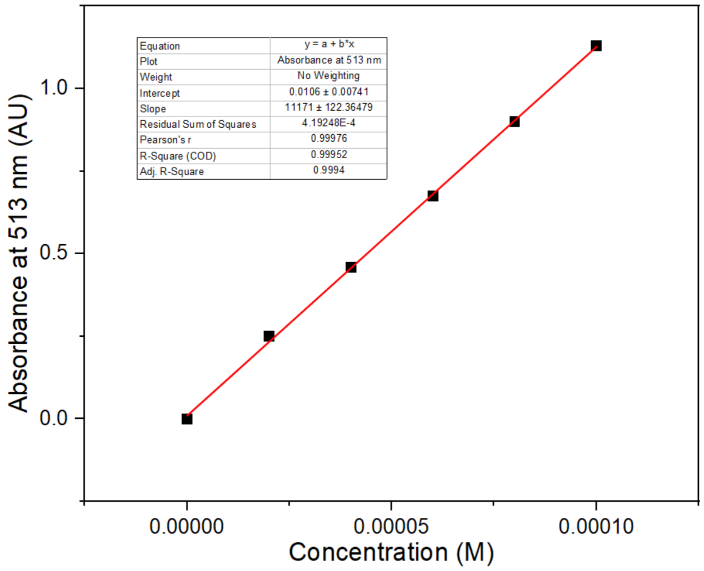

Figure S16. Determination of the molar extinction coefficient ε of $[Fe(phen)_3]^{2+}$ complex in PhCN.

- Yield of $[Fe(phen)_3]^{2+}$ complex formation

Sample preparation: A 60 µM sample of **Fc[6]CPP** in PhCN was prepared in a quartz cuvette by adding 114 µL of a fresh **Fc[6]CPP** solution in PhCN (1.00 mg, 1.56 µmol, in 1.00 mL) to 180 µl (500 equiv.) of 0.5 M phen solution in PhCN, and filled to volume of 3.00 mL with PhCN. The sample was stirred in ambient light for 24 h and filtered twice through a 0.22 µm PTFE syringe filter. The experiment was repeated four times (Table S4). The same procedure was applied to calculate the yield in H_2O/THF with $\varepsilon_{450\,nm}$ = 4,049 mol^{-1} cm^{-1} for **Fc[6]CPP** and $\varepsilon_{511\,nm}$ = 10,734 mol^{-1} cm^{-1} for $[Fe(phen)_3]^{2+}$ complex.

Example of calculation of yield of $[Fe(phen)_3]^{2+}$ complex formation (before and after filtration):

$$yield\ (before\ filtration) = \frac{concentration\ ([Fe(phen)_3]^{2+})\ at\ t = 24\ h}{concentration\ (Fc[6]CPP)\ at\ t = 0\ h} = \frac{\frac{A_{[Fe(phen)_3]^{2+}}}{\varepsilon_{513\ nm}}}{\frac{A_{Fc[6]CPP}}{\varepsilon_{450\ nm}}} = \frac{\frac{0.5334}{11{,}171}}{\frac{0.2731}{4{,}708}} = 82.31\ \%$$

$$yield\ (after\ filtration) = \frac{concentration\ ([Fe(phen)_3]^{2+})\ at\ t = 24\ h}{concentration\ (Fc[6]CPP)\ at\ t = 0\ h} = \frac{\frac{A_{[Fe(phen)_3]^{2+}}}{\varepsilon_{513\ nm}}}{\frac{A_{Fc[6]CPP}}{\varepsilon_{450\ nm}}} = \frac{\frac{0.4211}{11{,}171}}{\frac{0.2731}{4{,}708}} = 64.99\ \%$$

Table S4. Yield of $[Fe(phen)_3]^{2+}$ complex formation before and after filtration.

Experiment	Yield before filtration	Yield after filtration
**1**	82.31 %	64.99 %
**2**	75.91 %	60.40 %
**3**	83.77 %	66.64 %
**4**	79.32 %	74.70 %
**Average ± st. dev.**	80.3 ± 3.01 %	66.7 ± 5.16 %

5.4 Control experiments w/o light

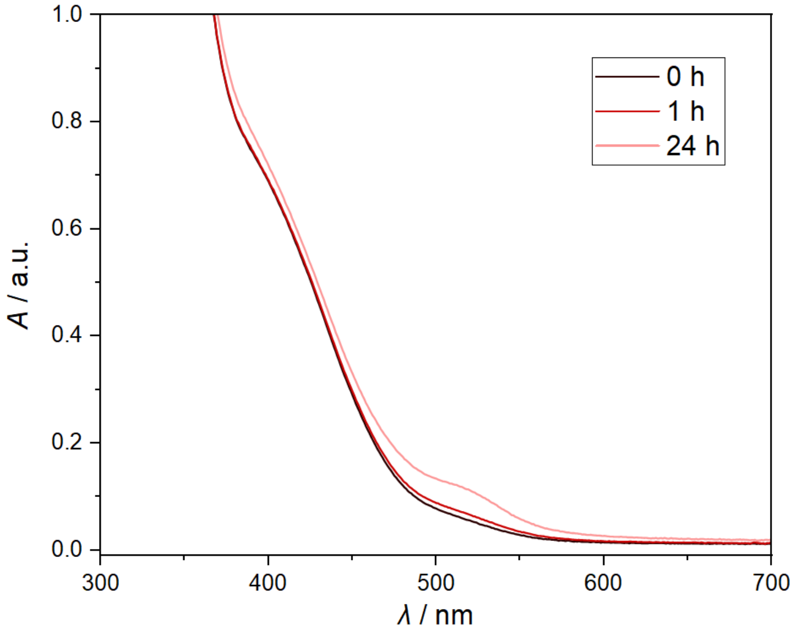

Figure S17. **Fc[6]CPP** in PhCN (≈ 60 µM) with phen (500 equiv.) stirred in dark at room temperature.

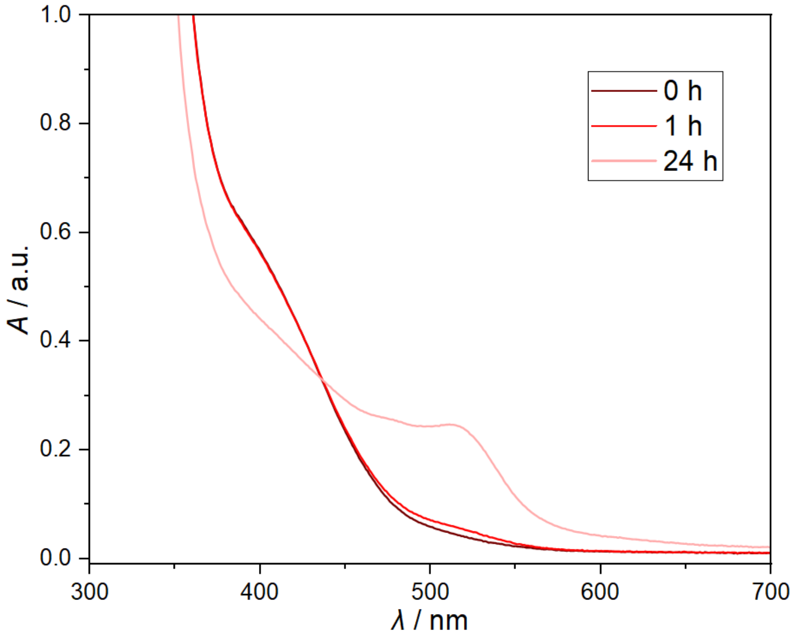

Figure S18. **Fc[6]CPP** in PhCN (≈ 60 µM) with phen (500 equiv.) stirred in dark at 70 °C.

5.6 Quantum yields of $[Fe(phen)_3]^{2+}$ complex formation

A solution of **Fc**, **pro-Fc[6]CPP** or **Fc[6]CPP** (see sample preparation below) in PhCN was stirred and left to equilibrate for 2–3 min in the dark at 20 °C. Afterward, the sample was irradiated with a beam of collimated light at 472 nm (Ø=7 mm) using a FC1-LED 470CG light source (Figure S19) coupled to a polymer optical fibre from Prizmatix.[13] UV–Vis spectra were recorded periodically using diode-array spectrophotometer. The radiant power (flux; Φ_e) of the light source was determined using calibrated Si-photodiode and optical power meter. The total irradiation time was selected to reach <10% conversion and to obtain 6-10 experimental points. The procedure was repeated 2–4 times. The quantum yield of formation Φ_{form} was calculated according to the equation:

$$\Phi_{form} = \frac{\Delta n_{form}}{\Delta n_{abs}^{p}} \quad \text{(Eq. 1)}$$

where Δn_{form} is the number of moles of $[Fe(phen)_3]^{2+}$ complex formed, calculated from the absorbance change at λ_{max} (513 nm), and Δn^{p}_{abs} is the number of moles of photons absorbed by the sample in the given time period calculated according to the equation:

$$\Delta n_{abs}^{p} = \frac{\int_0^t \int_0^\infty (1 - 10^{-A(\lambda,t)})\, I_\lambda^{em}\, d\lambda\, dt}{6.022 \times 10^{23}} \quad \text{(Eq. 2)}$$

where $A(\lambda,t)$ is the absorbance of the sample at the wavelength λ in time t, and I_λ^{em} is the photon flux of the LED source at the wavelength λ determined according to the equation:

$$I_\lambda^{em} = q_n(\lambda) \frac{\Phi_e}{\int_0^\infty \frac{hc}{\lambda} q_n(\lambda)\, d\lambda} \quad \text{(Eq. 3)}$$

where $q_n(\lambda)$ is the emission spectrum of the LED source provided by manufacturer (counts vs. wavelength) and Φ_e is the radiant power (flux) measured by the optical power meter.

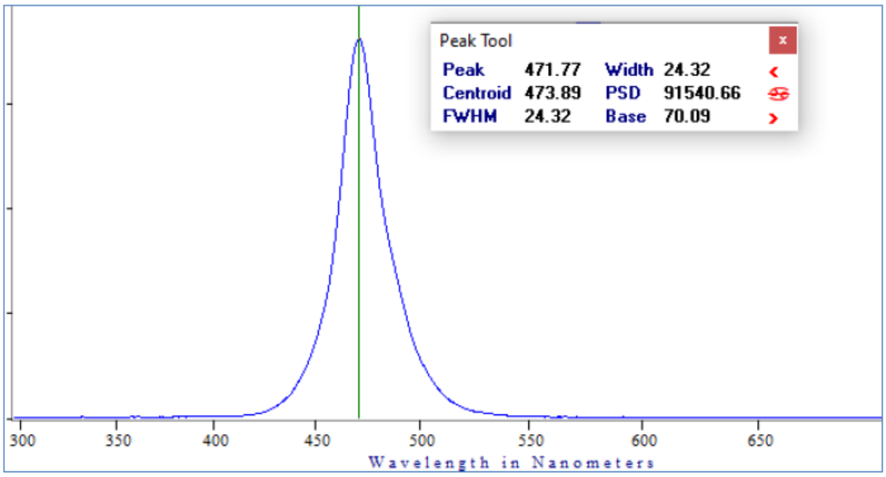

Figure S19. Emission spectrum of the FC1-LED 470CG light source (obtained from Prizmatix[13]).

Sample preparation:

Fc: A 40 μM solution of **Fc** in PhCN was prepared in a quartz cuvette by adding 22 μL (1 equiv.) of 5.38 mM **Fc** solution in PhCN to 120 μL (500 equiv.) of 0.5 M phen solution in PhCN, followed by PhCN (total volume = 3.00 mL). The sample was prepared covered in an aluminum foil and then irradiated (472 nm, Φ_e = 130.2 mW) for ~1.5 h while stirring. The experiment was repeated four times.

Pro-Fc[6]CPP: A 40 μM solution of **pro-Fc[6]CPP** in PhCN was prepared in a quartz cuvette by adding 92 μL (1 equiv.) of 1.31 mM **pro-Fc[6]CPP** solution in PhCN to 120 μL (500 equiv.) of 0.5 M phen solution in PhCN, followed by PhCN (total volume = 3.00 mL). The sample was prepared covered in an aluminum foil and then irradiated (472 nm, Φ_e = 130.2 mW) for 0.5 h while stirring. The experiment was repeated four times.

Fc[6]CPP: A 40 μM solution of **Fc[6]CPP** in PhCN was prepared in a quartz cuvette by adding 76 μL (1 equiv.) of 1.56 mM **Fc[6]CPP** solution in PhCN to phen solution in PhCN and PhCN (total volume = 3.00 mL):

- 5 equiv.: 20 μL of 0.0296 M phen + 2.904 mL PhCN
- 10 equiv.: 40 μL of 0.0296 M phen + 2.884 mL PhCN (effect of oxygen)
- 25 equiv.: 100 μL of 0.0296 M phen + 2.824 mL PhCN
- 100 equiv.: 24 μL of 0.5 M phen + 2.900 mL PhCN
- 250 equiv.: 60 μL of 0.5 M phen + 2.864 mL PhCN
- 500 equiv.: 120 μL of 0.5 M phen + 2.804 mL PhCN

The sample was prepared in a cuvette covered in aluminum foil and then irradiated (472 nm, Φ_e = 9.86 – 46.4 mW) for 1 or 2 min while stirring. The experiment was repeated four or two times. The same procedure was applied to calculate the quantum yield in H_2O/THF.

Table S5. Quantum yields of $[Fe(phen)_3]^{2+}$ complex formation from **Fc**, **pro-Fc[6]CPP** and **Fc[6]CPP** in PhCN in the presence of phen upon irradiation with a 472 nm light source.

Compound	phen (equiv.)	Φ_e *(mW)*	Φ_{form}	Average	STD[a]
**Fc**	500	130.2	$1.70 \cdot 10^{-5}$	$1.52 \cdot 10^{-5}$	$2.62 \cdot 10^{-6}$
**Fc**	500	130.2	$1.11 \cdot 10^{-5}$		
**Fc**	500	130.2	$1.79 \cdot 10^{-5}$		
**Fc**	500	130.2	$1.48 \cdot 10^{-5}$		
**Fc**	500	130.2	$1.45 \cdot 10^{-5}$ [b]	$2.02 \cdot 10^{-5}$ [b]	n.d.[c]
**Fc**	500	130.2	$2.42 \cdot 10^{-5}$ [b]		
**Fc**	500	130.2	$2.50 \cdot 10^{-5}$ [b]		
**Fc**	500	130.2	$1.71 \cdot 10^{-5}$ [b]		
**pro-Fc[6]CPP**	500	130.2	$5.39 \cdot 10^{-5}$	$4.91 \cdot 10^{-5}$	$3.99 \cdot 10^{-6}$
**pro-Fc[6]CPP**	500	130.2	$5.20 \cdot 10^{-5}$		
**pro-Fc[6]CPP**	500	130.2	$4.40 \cdot 10^{-5}$		
**pro-Fc[6]CPP**	500	130.2	$4.66 \cdot 10^{-5}$		
**Fc[6]CPP**	5	46.4	0.0017	$1.57 \cdot 10^{-3}$	$1.19 \cdot 10^{-4}$
**Fc[6]CPP**	5	46.4	0.00138		
**Fc[6]CPP**	5	46.4	0.00155		
**Fc[6]CPP**	5	46.4	0.00163		
**Fc[6]CPP**	25	25.6	0.00781	$6.96 \cdot 10^{-3}$	$8.55 \cdot 10^{-4}$
**Fc[6]CPP**	25	33.2	0.0061		
**Fc[6]CPP**	100	9.86	0.0357	$3.41 \cdot 10^{-2}$	$3.12 \cdot 10^{-3}$

**Fc[6]CPP**	100	9.86	0.0371		
**Fc[6]CPP**	100	9.86	0.0347		
**Fc[6]CPP**	100	9.86	0.0289		
**Fc[6]CPP**	250	9.86	0.0483	$5.27 \cdot 10^{-2}$	$4.35 \cdot 10^{-3}$
**Fc[6]CPP**	250	9.86	0.057		
**Fc[6]CPP**	500	9.86	0.0601	$6.00 \cdot 10^{-2}$	$4.88 \cdot 10^{-3}$
**Fc[6]CPP**	500	9.86	0.0669		
**Fc[6]CPP**	500	9.86	0.0531		
**Fc[6]CPP**	500	9.86	0.0597		

[a] STD = standard deviation of the mean. [b] Calculated manually using the same datasets measured for the samples of **Fc**, but assuming the lightsource was monochromatic (472 nm) and the absorbed photons were obtained from Lambert-Beer law to verify the validity of the used script, which employed equations 1–3. [c] n.d. = not determined.

Representative examples of the obtained slopes (= quantum yields):

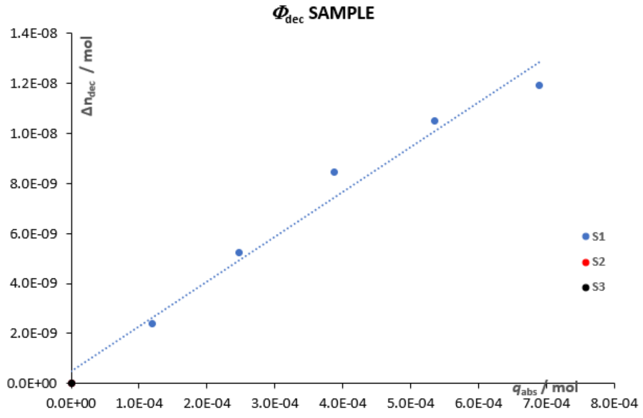

Figure S20. **Fc** + phen (500 equiv.).

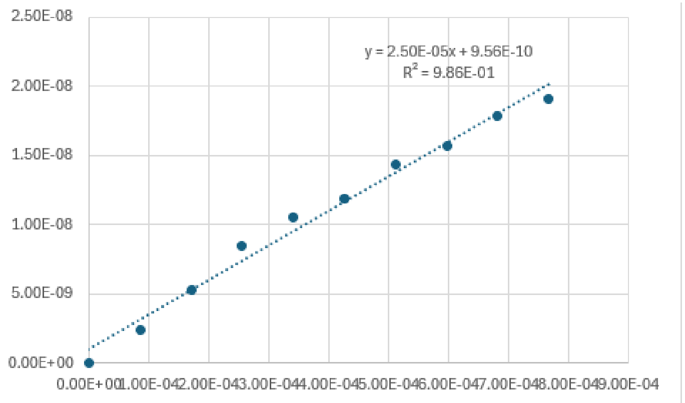

Figure S21. **Fc** + phen (500 equiv.) assuming the lightsource was monochromatic (472 nm) and the absorbed photons calculated from Lambert-Beer law to verify the validity of the used computer script based on equations 1–3.

Φ_{form} = 5.39E-05 ± 0.0E+00

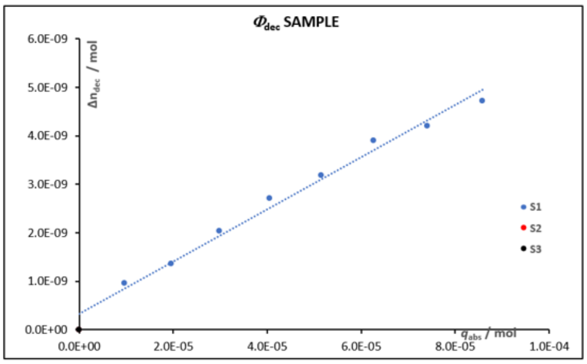

Figure S22. **Pro-Fc[6]CPP** + phen (500 equiv.). Note that the error is not reported because a single dataset is displayed.

Φ_{form} = 1.55E-03 ± 0.0E+00

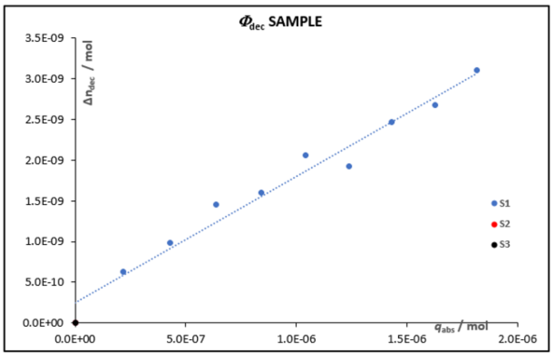

Figure S23. **Fc[6]CPP** + phen (5 equiv.). Note that the error is not reported because a single dataset is displayed.

Φ_{form} = 7.81E-03 ± 0.0E+00

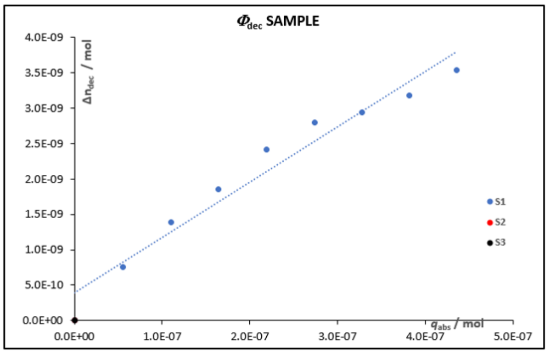

Figure S24. **Fc[6]CPP** + phen (25 equiv.). Note that the error is not reported because a single dataset is displayed.

Φ_{form} = 3.57E-02 ± 0.0E+00

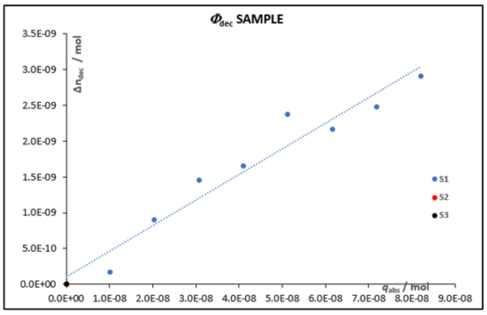

Figure S25. **Fc[6]CPP** + phen (100 equiv.). Note that the error is not reported because a single dataset is displayed.

Φ_{form} = 4.83E-02 ± 0.0E+00

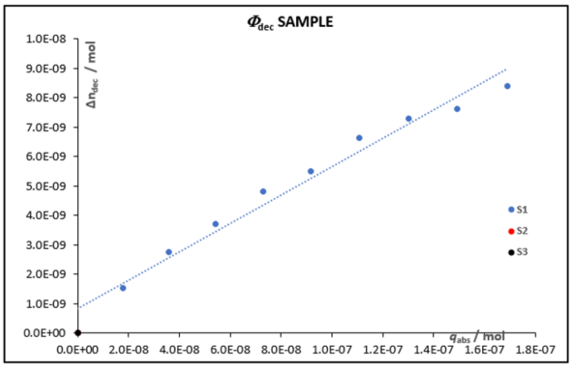

Figure S26. **Fc[6]CPP** + phen (250 equiv.). Note that the error is not reported because a single dataset is displayed.

Φ_{form} = 6.01E-02 ± 0.0E+00

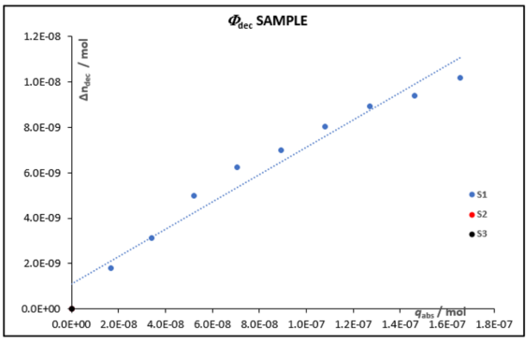

Figure S27. **Fc[6]CPP** + phen (500 equiv.). Note that the error is not reported because a single dataset is displayed.

5.7 **Quantum yield simulations**

Assuming that $[Fe(phen)_3]^{2+}$ formation from **Fc[6]CPP** occurs via its triplet state, the quantum yield of $[Fe(phen)_3]^{2+}$ complex formation Φ_{form} can be written as:

$$\Phi_{form} = \Phi_{ISC} \cdot \Phi_{phen}$$

where Φ_{ISC} (= 0.085)[14] is the quantum yield of intersystem crossing reported for **Fc** ($E_T \sim$ 40 kcal mol^{-1}) and Φ_{phen} is the quantum yield of quenching of the excited T_1 state by phen which can be defined (in the presence of oxygen) as:

$$\Phi_{phen} = \frac{k_{diff} \cdot [phen]}{\frac{1}{\tau_{T_1}} + k_{diff} \cdot [phen] + k_{diff} \cdot [O_2]}$$

where k_{diff} (≈ 5 × 10^{10} M^{-1} s^{-1}) is the approximate diffusion rate constant in PhCN, τ_{T_1}(= 9 × 10^{-8} s)[14] is the lifetime of the T_1 state reported for **Fc**. Thus, Φ_{form} can be defined as:

$$\Phi_{form} = \Phi_{ISC} \cdot \frac{k_{diff} \cdot [phen]}{\frac{1}{\tau_{T_1}} + k_{diff} \cdot [phen] + k_{diff} \cdot [O_2]}$$

This equation was used to calculate Φ_{form} from **Fc[6]CPP** in the presence ($[O_2]$ ≈ 2.5 × 10^{-3} M) and the absence of oxygen ($[O_2]$ = 0 M) for concentrations of phenanthroline ranging from 2 × 10^{-4} M to 2 × 10^{-2} M (corresponding to 5 to 500 equiv. in the experiments performed in section 5.6).

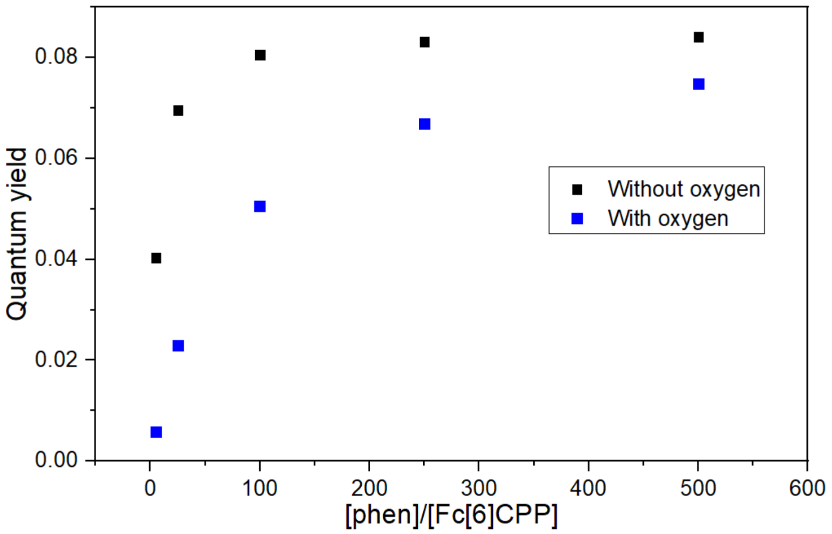

Figure S28. Simulations of quantum yields of $[Fe(phen)_3]^{2+}$ formation from **Fc[6]CPP** and different equivalents of phen in the absence and the presence of oxygen.

The quantum yields of $[Fe(phen)_3]^{2+}$ complex formation from **Fc[6]CPP** with 10 or 25 equiv. of phen in PhCN in the presence and the absence of oxygen were determined using the procedure described in section 5.6. Prior to the measurement, the sample was either oxygenated by bubbling with oxygen or degassed by bubbling with nitrogen for 20 min. The sample was then irradiated (472 nm) for 20 sec while stirring. Each experiment was repeated at least four times.

5.8 Mechanistic insights

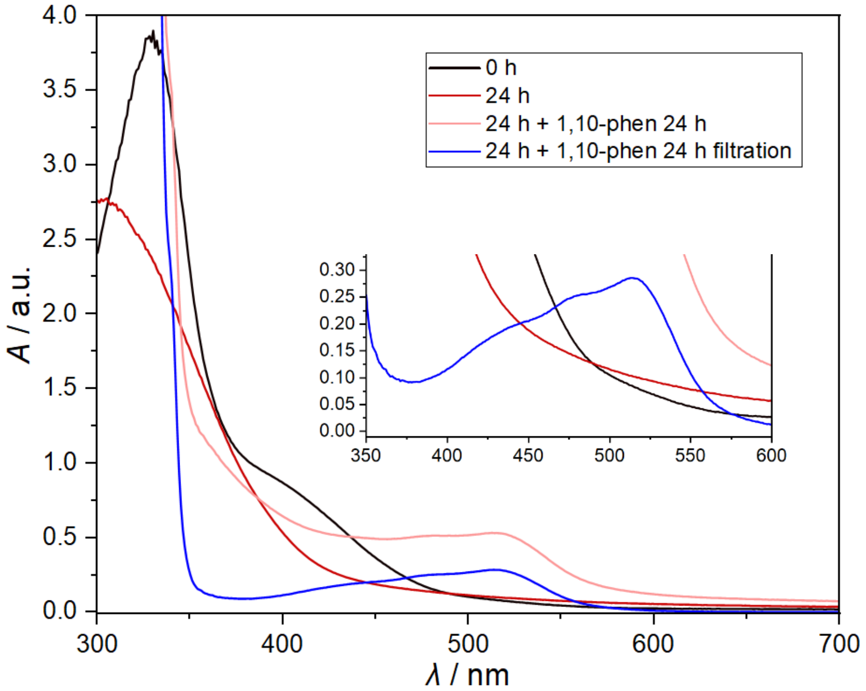

Figure S29. **Fc[6]CPP** in PhCN (≈ 60 µM) stirred in ambient light for 24 h, followed by addition of phen (500 equiv.) and stirring in ambient light.

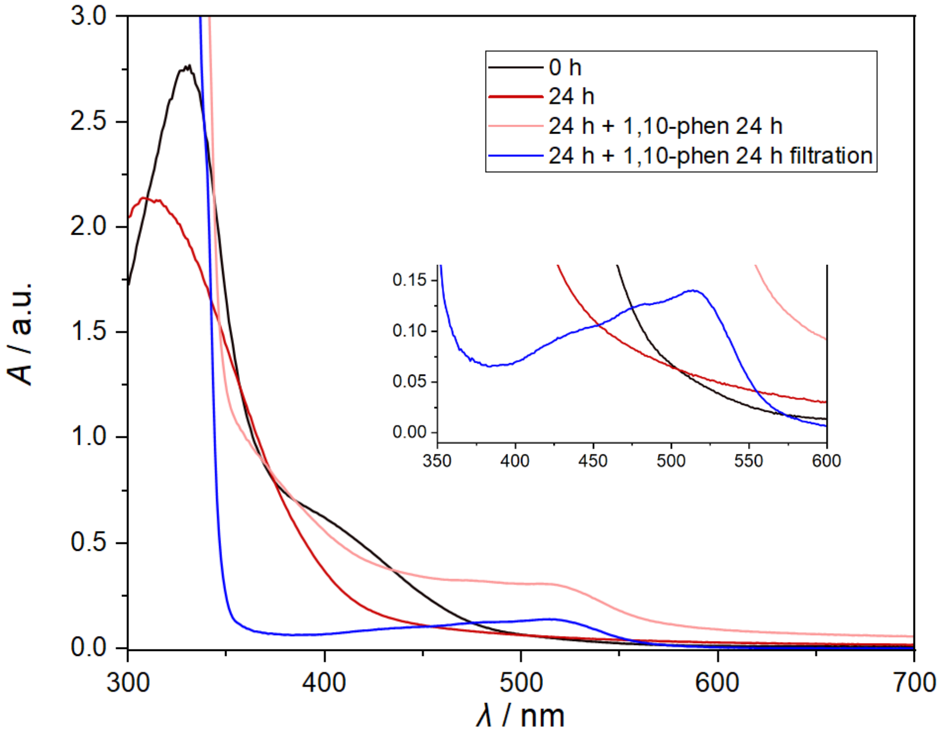

Figure S30. **Fc[6]CPP** in PhCN (≈ 60 µM) stirred in ambient light for 24 h, followed by addition of phen (500 equiv.) and stirring in dark.

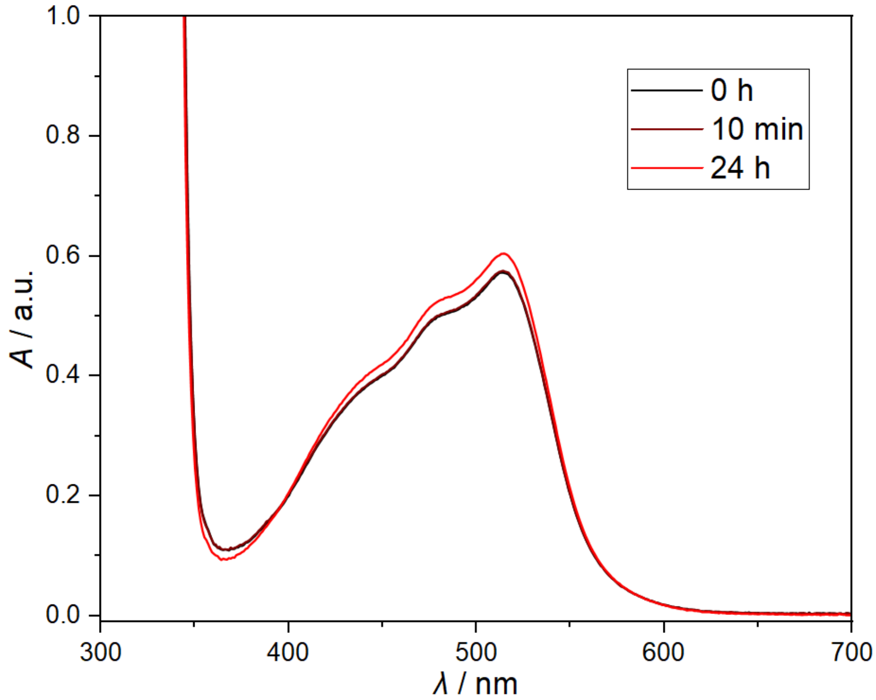

Figure S31. $Fe(OTf)_2$ + phen (500 equiv.) in PhCN (≈ 60 μM) stirred in ambient light for 24 h.

5.9 Experiments in THF/H_2O

The experiments requiring irradiation with green light (λ_{LED} = 525 nm, FWHM = 35 nm) were done using the PR160L-525 LED lamp (25 % intensity) purchased from Kessil.[15] The samples were placed 20 cm away from the light source.

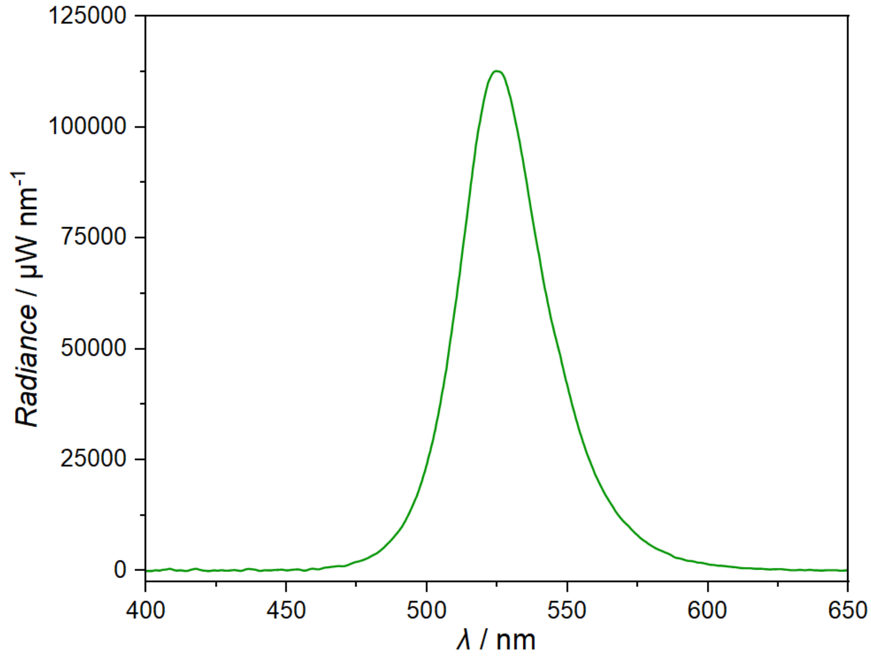

Figure S32. Emission spectrum of the PR160L-525 LED lamp (obtained from Kessil[15]).

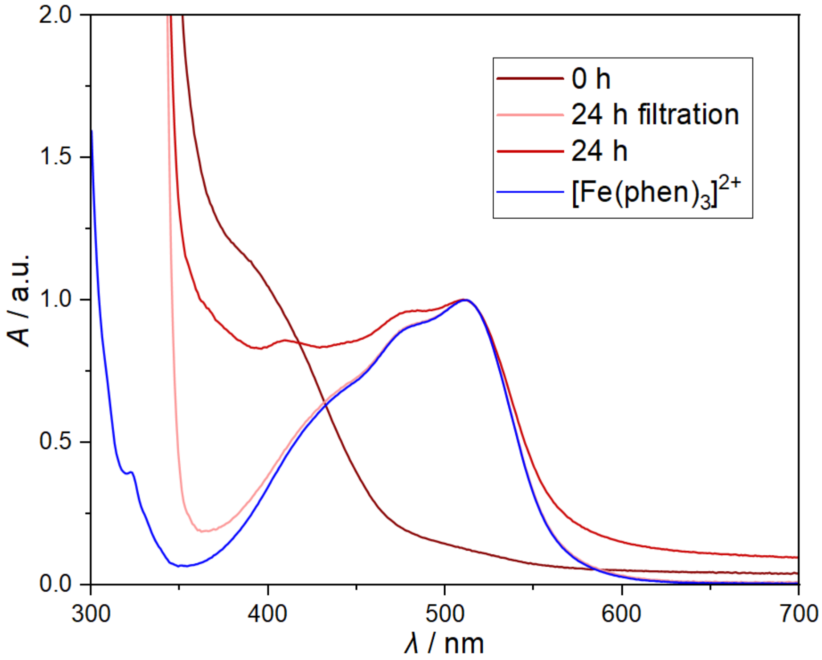

Figure S33. Normalized absorption spectra of **Fc[6]CPP** in H_2O/THF (*v*/*v* = 1:1, c ≈ 60 µM, phen: 500 equiv.) stirred in green light (λ_{LED} = 525 nm) for 24 h (red: unfiltered, light red: filtered) and independently prepared $[Fe(phen)_3]^{2+}$ (blue). The spectrum of the solution at 0 h (dark red) was scaled such that the absorbance at 400 nm matched the absorbance of $[Fe(phen)_3]^{2+}$ with equal concentration at its ε_{max} = 511 nm.

6. DFT calculations

6.1 General remarks

All calculations were performed with Gaussian 16[16] (release C.02) software. The crystal structures of **pro-Fc[6]CPP** and **Fc[6]CPP** were used as a starting point and geometries were optimized at D3-B3LYP/6-31+g(d)/LanL2DZ(Fe) level of theory. Effective core potential was used for iron atoms. For triplet states, we needed to check and eventually reoptimize the triplet wavefunction. The stable wavefunction was then used to optimize the geometries. The minima on potential energy surface were confirmed by subsequent frequency calculations.

Table S6. Geometrical parameters of the studied species as compared to crystal structures.

Molecule	Method	Multiplicity	α / °	δ / °	d / Å[a]
**pro-Fc[6]CPP**	X-ray	-	0.67	179.75	10.961
	D3-B3LYP	1	0.91	179.73	11.097
		3	1.01	179.46	11.128
**Fc[6]CPP**	X-ray	-	10.62	172.84	7.004
	D3-B3LYP	1	6.97	175.61	6.920
		3	12.05	173.43	7.236

[a]Diameter of the cavity was determined as the distance between centroids of the cyclohexadiene moieties for **pro-Fc[6]CPP** and corresponding phenylenes for **Fc[6]CPP**.

6.2 Strain energy

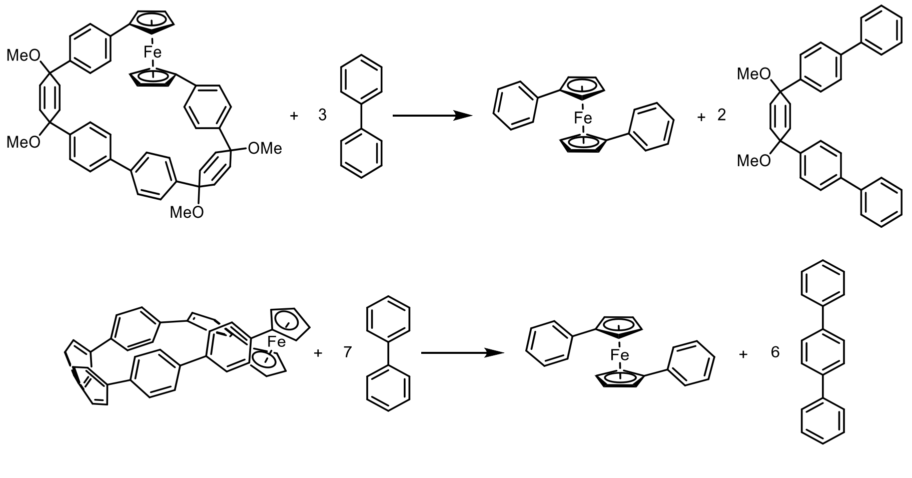

Scheme S1. Homodesmotic reactions used to calculate strain energy of the **pro-Fc[6]CPP** (top) and **Fc[6]CPP** (bottom).

Table S7. Strain energies in kcal mol^{-1} calculated using 6-31++g(d)/LanL2DZ(Fe) basis sets and the corresponding functional.

Compound	D3-B3LYP	wB97XD
**pro-Fc[6]CPP**	13.7	11.4
**Fc[6]CPP**	82.6	85.5

6.3 Frontier molecular orbitals

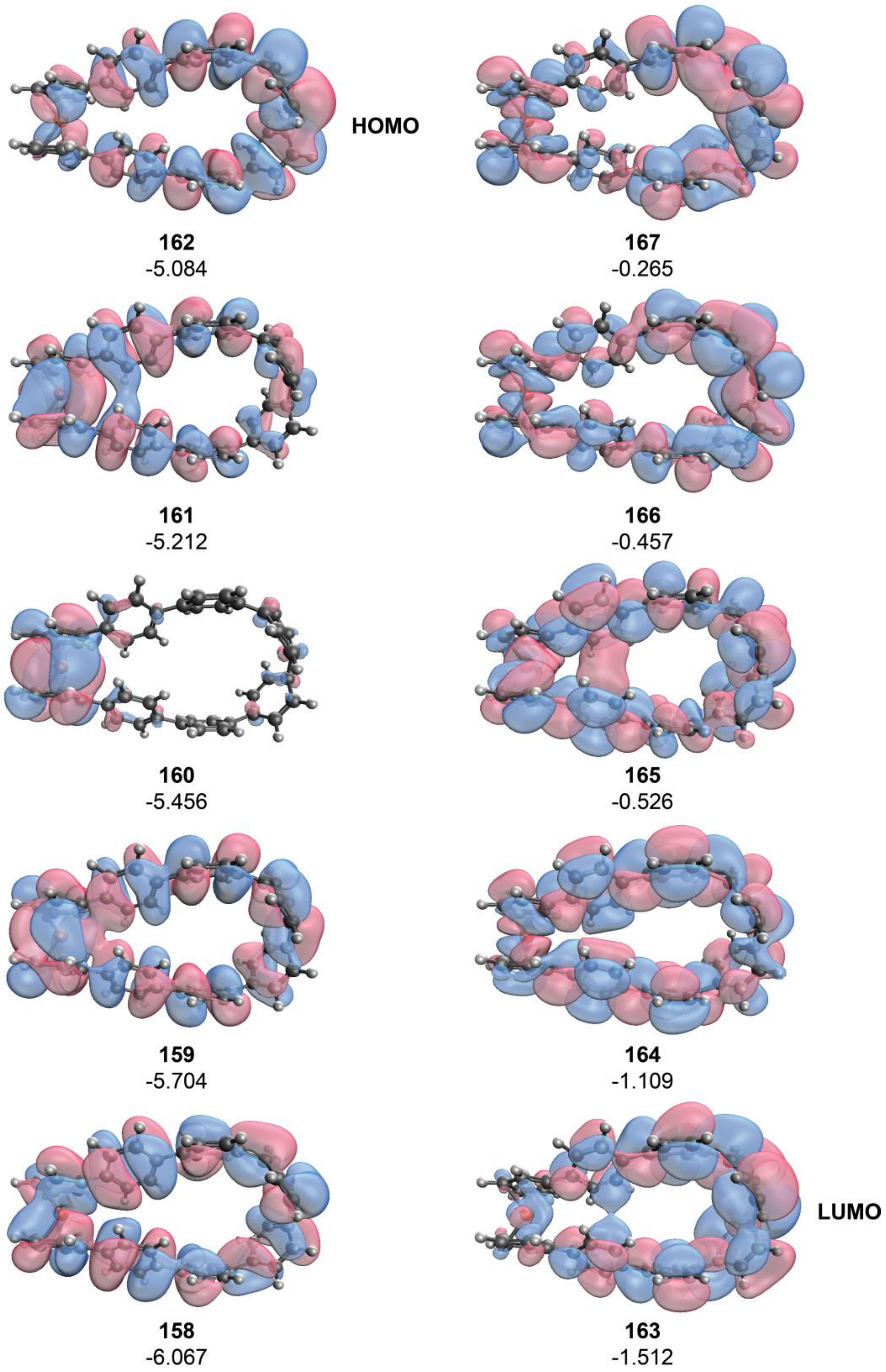

Figure S34. Frontier molecular orbitals of **Fc[6]CPP** (singlet) with orbital number and their energy in eV at D3-B3LYP/6-31g(d)/LanL2DZ(Fe) level of theory. Note that the energies of the virtual MOs are only approximate.

6.4 Spin density plot

Spin density of triplet **Fc[6]CPP** was calculated at (U)-D3-B3LYP/6-31g(d)/LanL2DZ(Fe) level of theory.

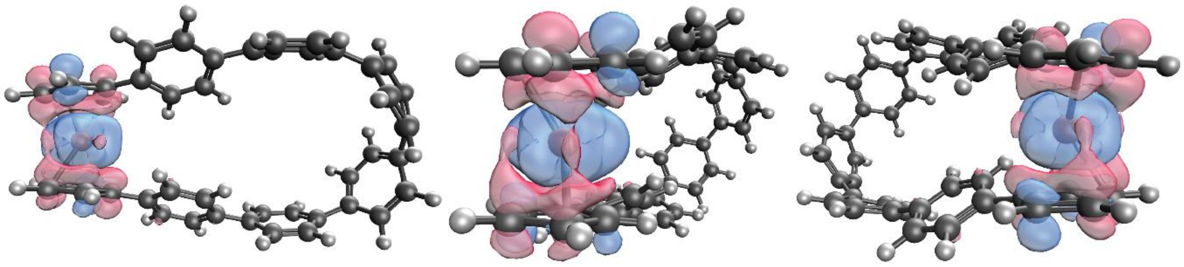

Figure S35. Spin density distribution in triplet **Fc[6]CPP** (isosurface value = 0.02).

6.5 Time dependent DFT calculations

Natural transition orbitals (NTOs) were plotted at the TD-CAM-B3LYP/6-31g(d)/LanL2DZ(Fe) level of theory (isosurface value = 0.02).

Table S8. The first 10 transitions of **Fc[6]CPP** at TD-CAM-B3LYP/6-31g(d)/LanL2DZ(Fe) level of theory.

Transition	E / eV	λ / nm	f	Orbital contributions[a]
**1**	1.93	643	0.0007	159->177 (14%)
**2**	1.94	640	0.0008	159->172 (24%)<br>160->177 (15%)
**3**	2.46	503	0.0017	149->177 (12%)<br>160->172 (26%)
**4**	2.49	499	0	149->172 (23%)<br>159->172 (20%)
**5**	3.40	365	0.0001	149->177 (15%)<br>160->172 (16%)
**6**	3.46	358	0.0007	149->172 (43%)
**7**	3.51	353	0.1661	162->163 (85%)
**8**	4.29	289	1.2343	161->163 (19%)<br>162->164 (38%)<br>162->166 (18%)
**9**	4.46	278	0.5605	155->163 (11%)<br>161->163 (23%)<br>162->166 (21%)<br>162->169 (10%)
**10**	4.52	274	0.411	161->164 (47%)<br>162->165 (13%)

[a]Only the contributions larger than 10% or the highest contributing transitions are listed.

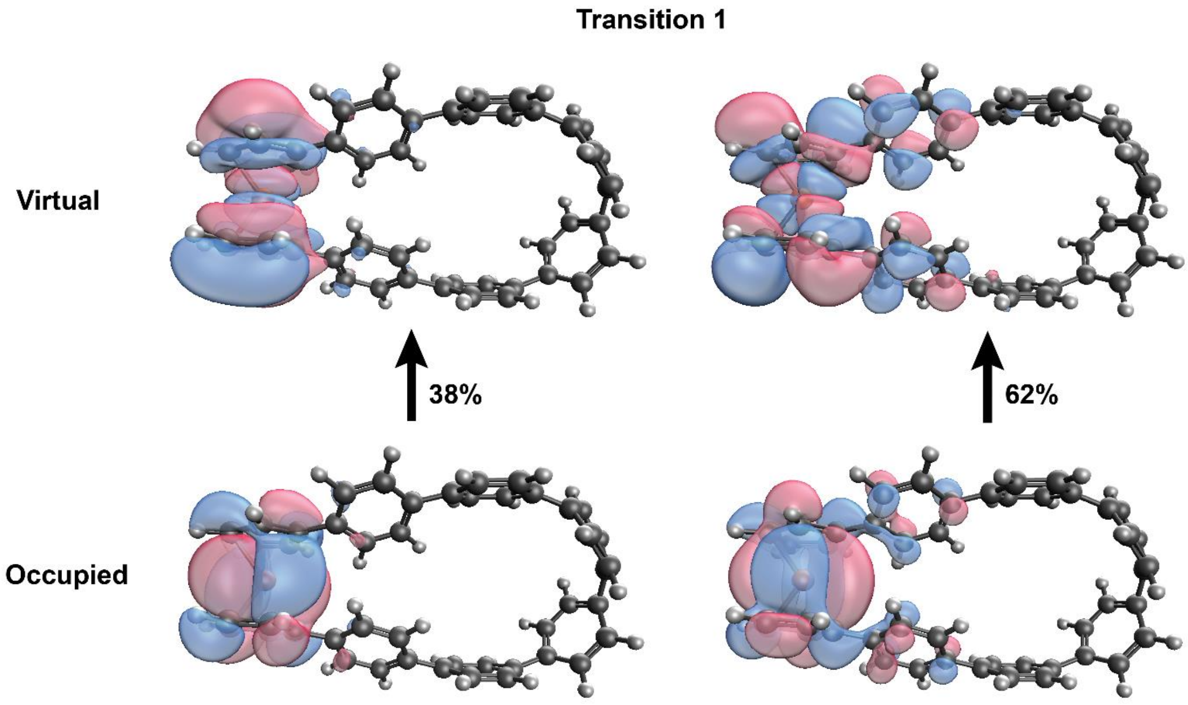

Figure S36. NTO of the $S_0 \rightarrow S_1$ transition (E = 1.93 eV, f = 0.0007) of **Fc[6]CPP**.

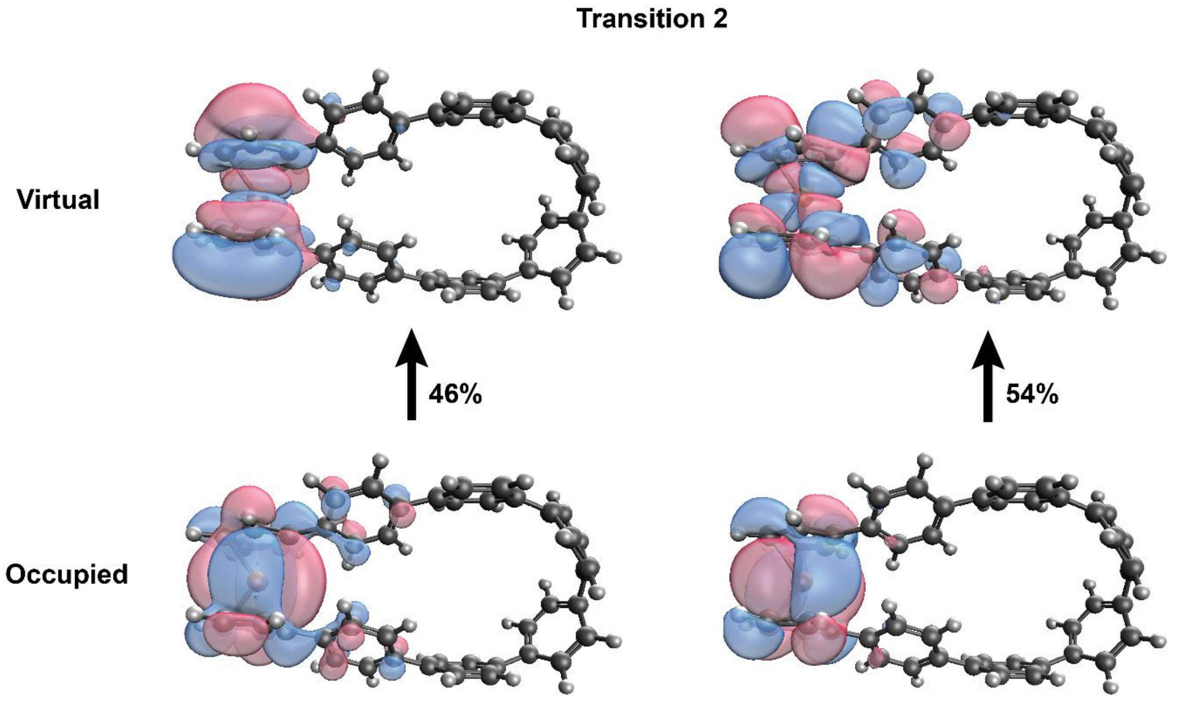

Figure S37. NTO of the $S_0 \rightarrow S_2$ transition (E = 1.94 eV, f = 0.0008) of **Fc[6]CPP**.

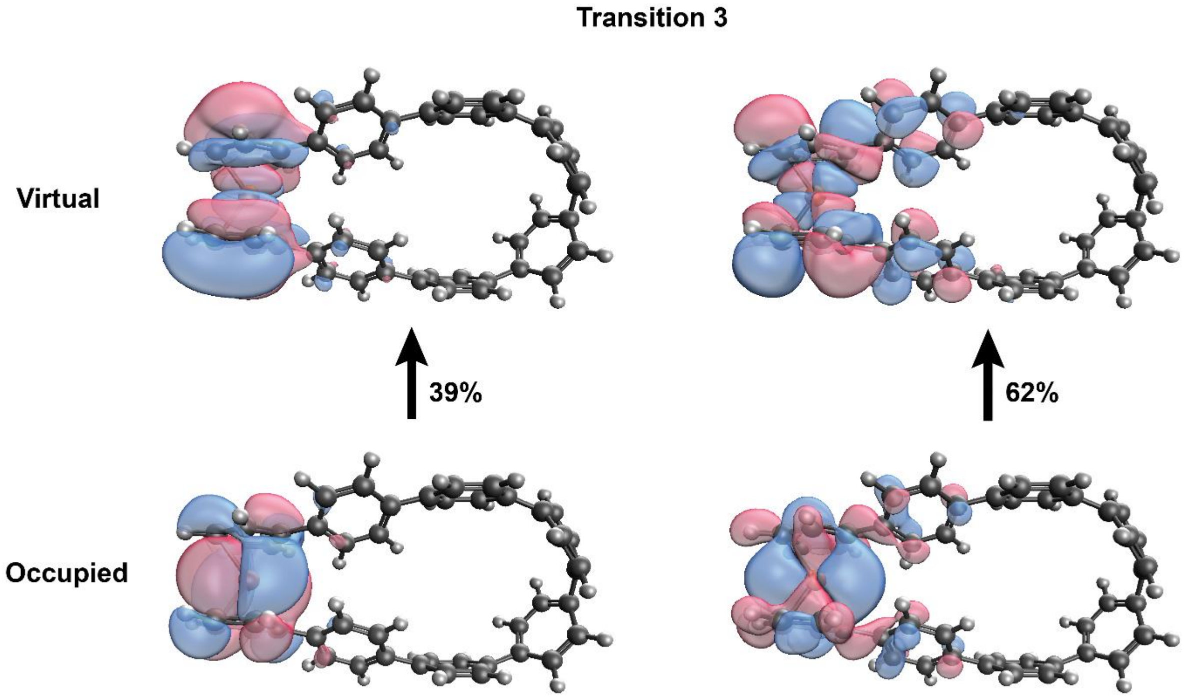

Figure S38. NTO of the $S_0 \rightarrow S_3$ transition (E = 2.46 eV, f = 0.0017) of **Fc[6]CPP**.

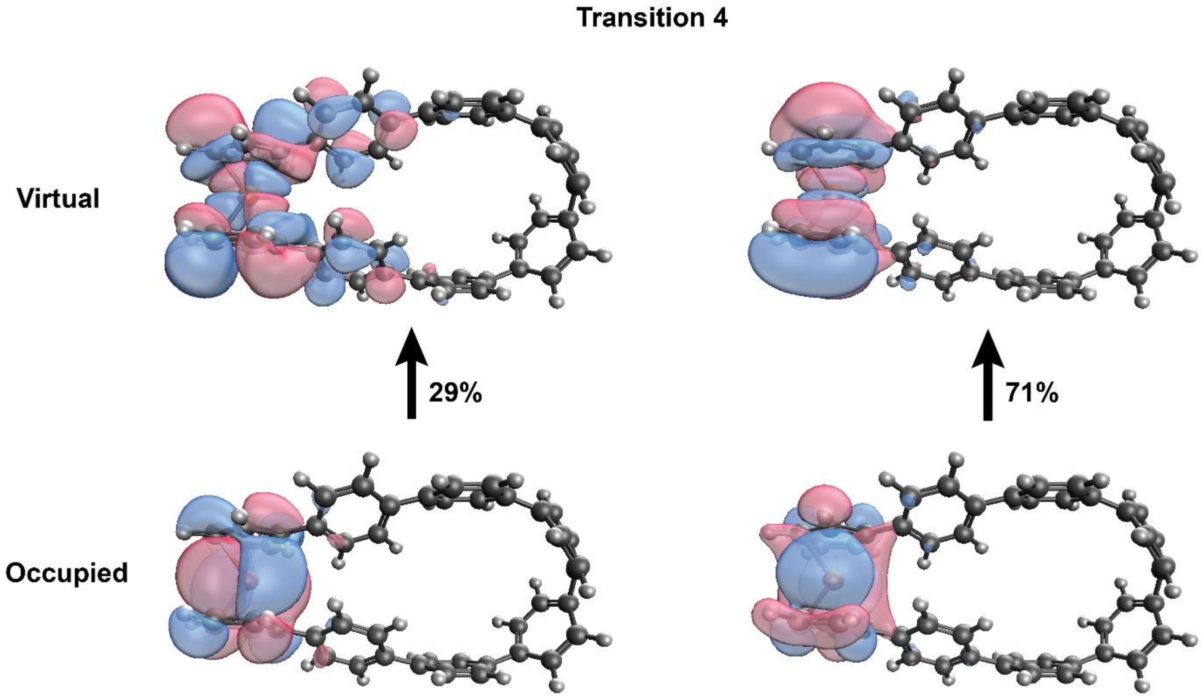

Figure S39. NTO of the $S_0 \rightarrow S_4$ transition (E = 2.49 eV, f = 0) of **Fc[6]CPP**.

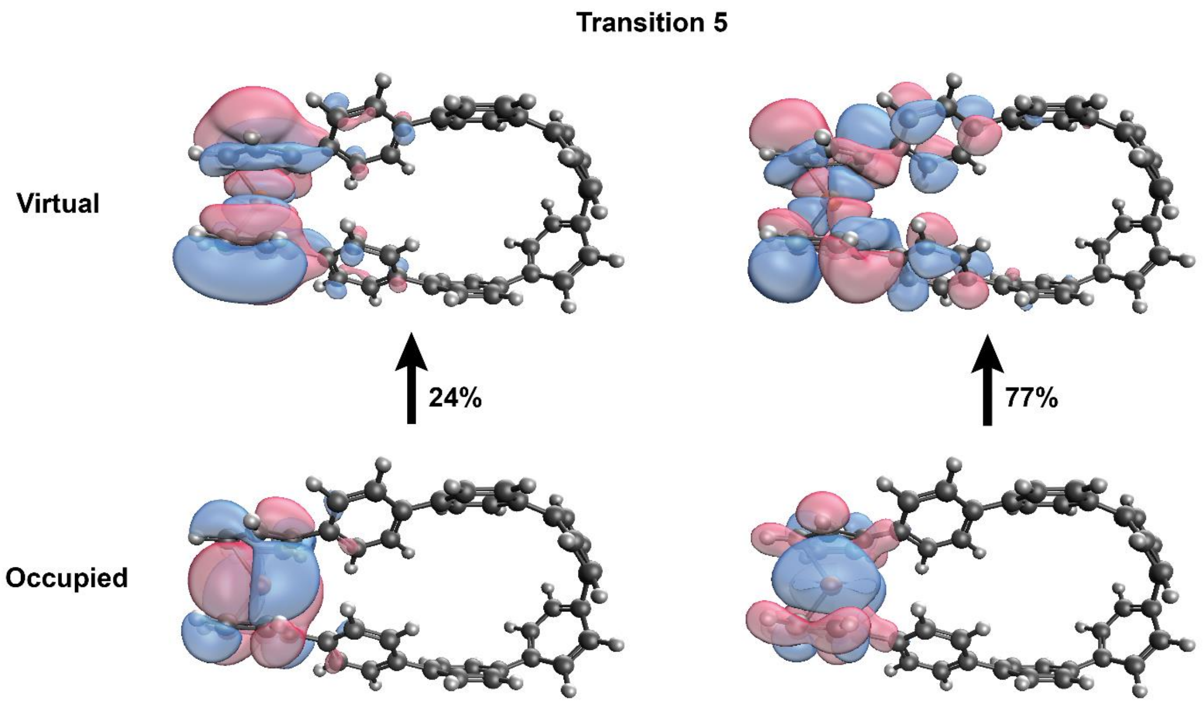

Figure S40. NTO of the $S_0 \rightarrow S_5$ transition (E = 3.40 eV, f = 0.0001) of **Fc[6]CPP**.

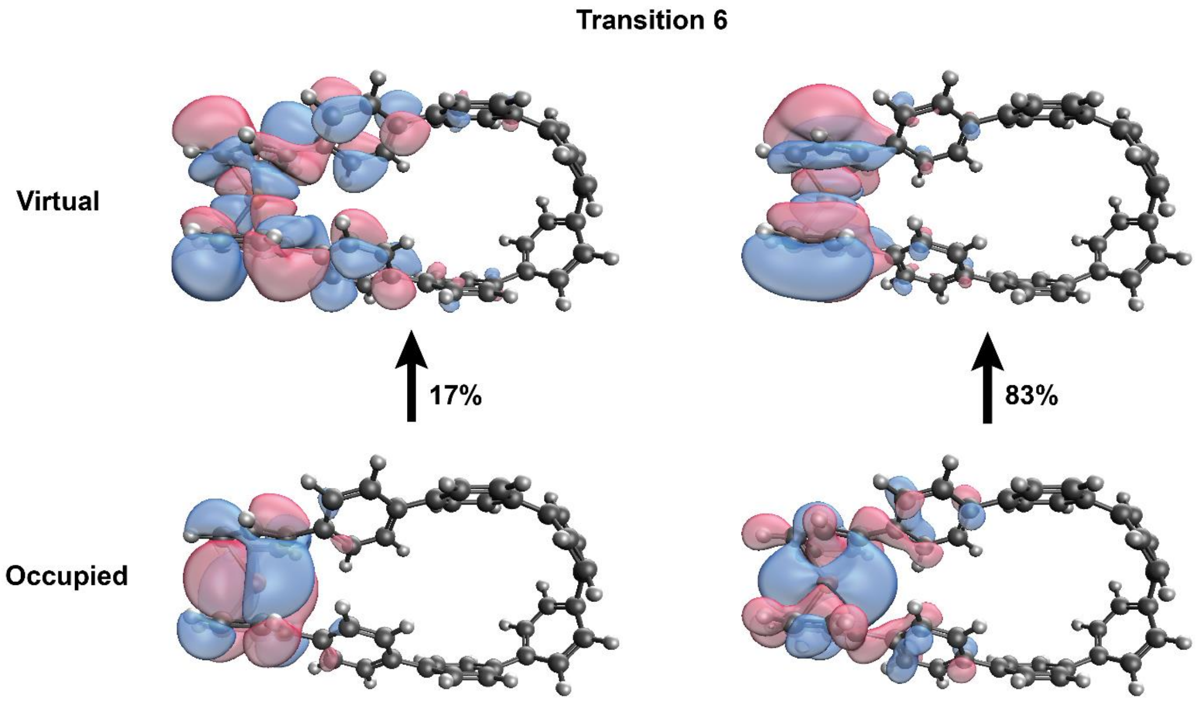

Figure S41. NTO of the $S_0 \rightarrow S_6$ transition (E = 3.46 eV, f = 0.0007) of **Fc[6]CPP** with their occupancies.

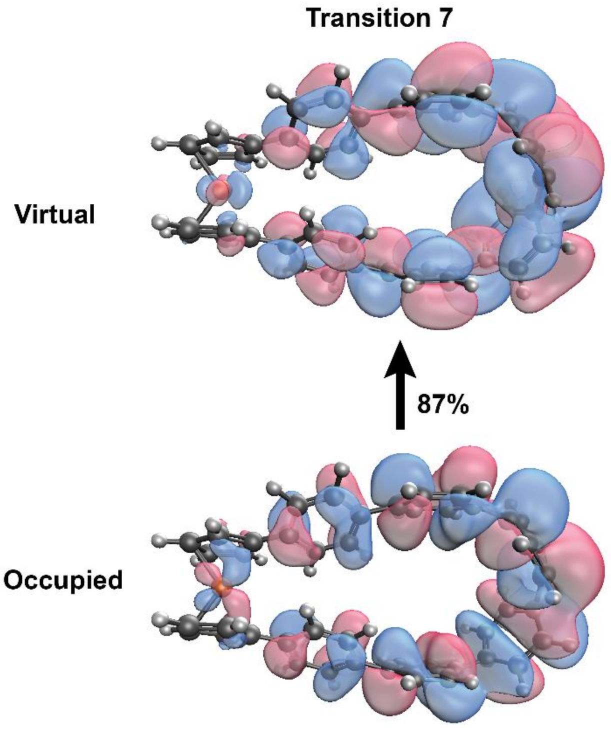

Figure S42. NTO of the $S_0 \rightarrow S_7$ transition (E = 3.51 eV, f = 0.1661) of **Fc[6]CPP** with their occupancies.

7. ^{1}H and [13]NMR spectra

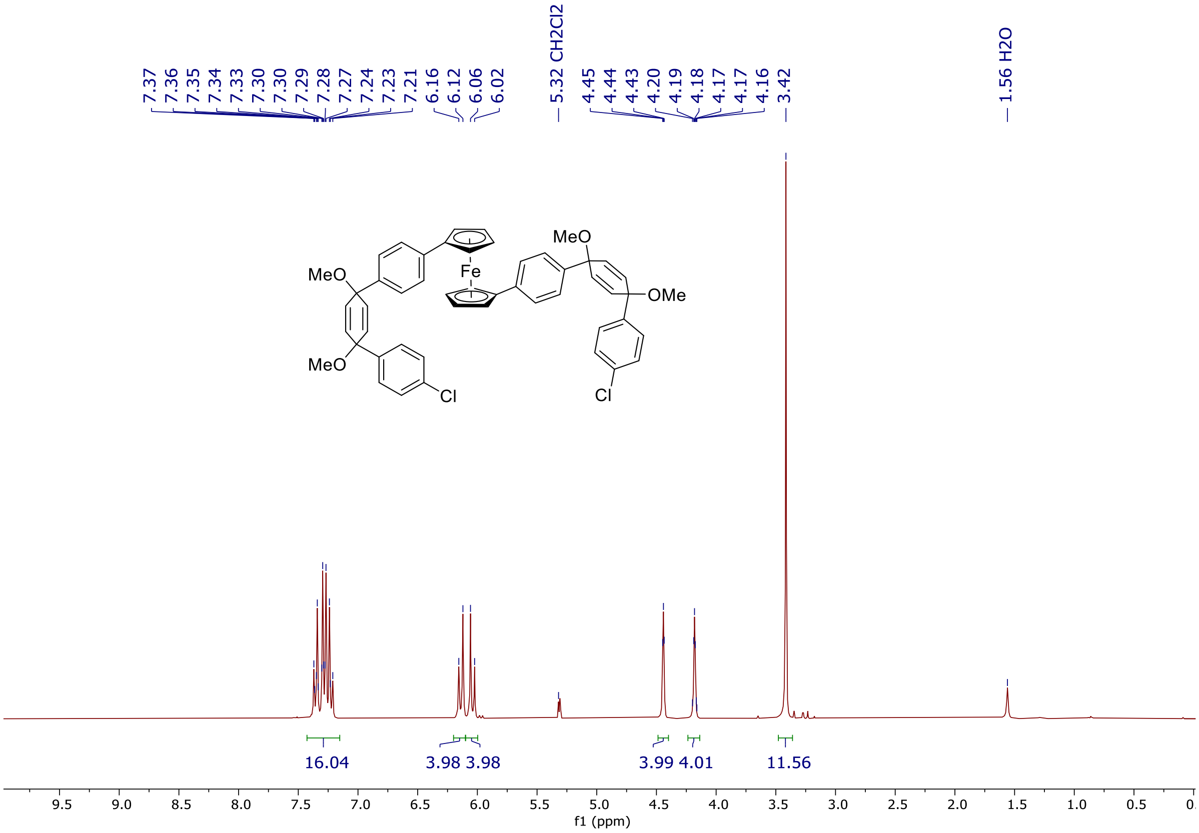

^{1}H NMR spectrum of compound **2** in CD_2Cl_2 (300 MHz).

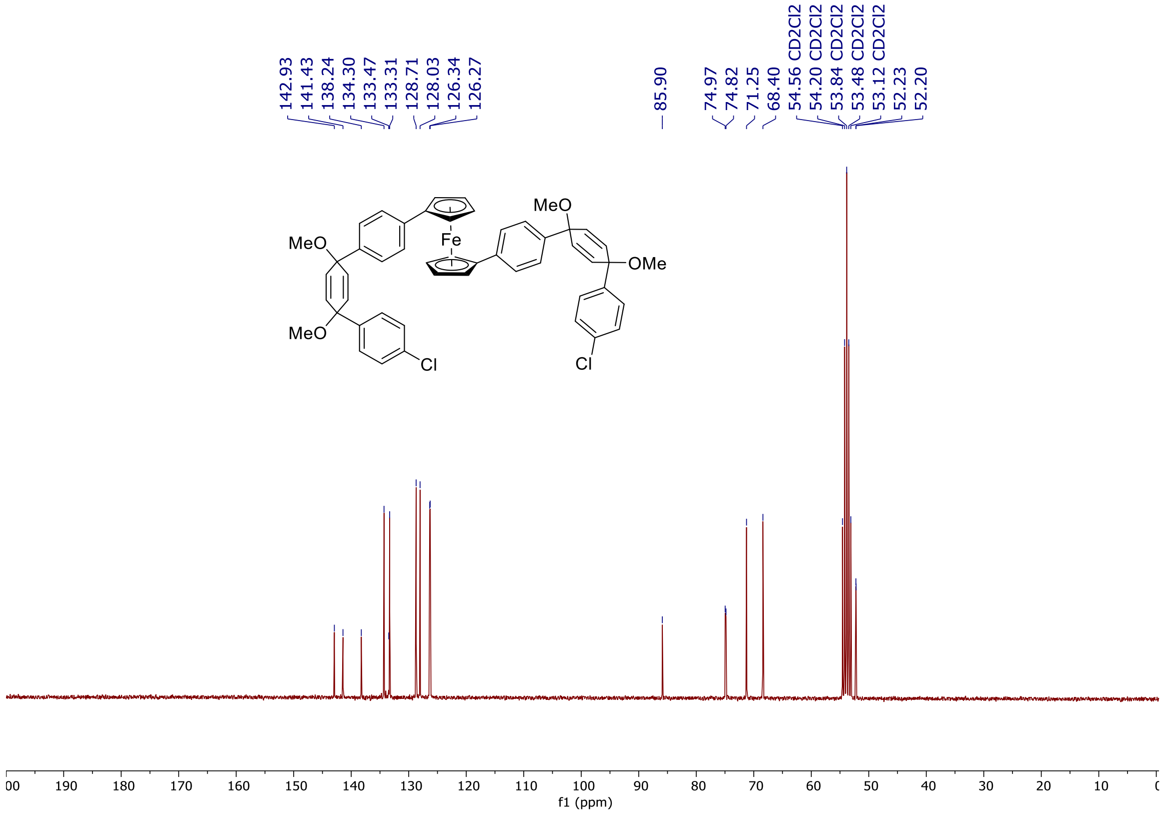

^{13}C NMR spectrum of compound **2** in CD_2Cl_2 (75 MHz).

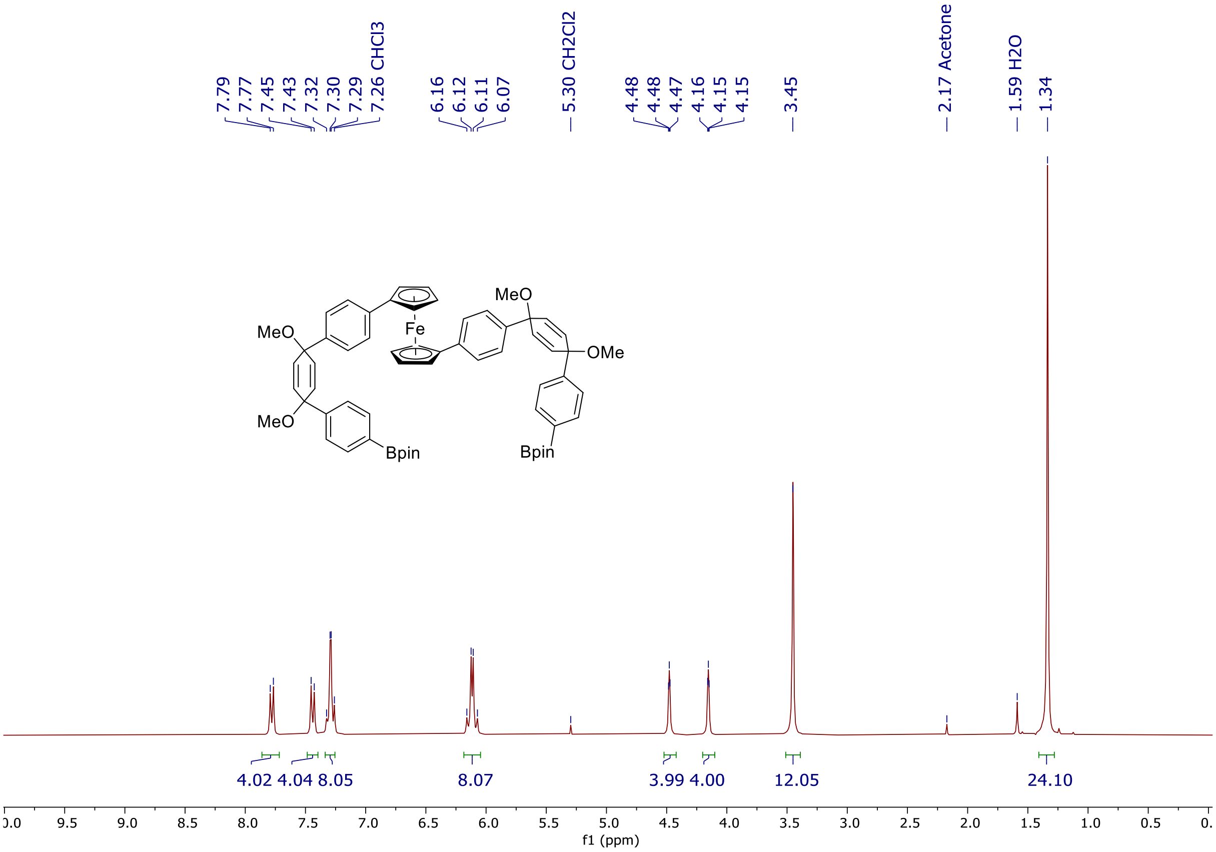

^{1}H NMR spectrum of compound **3** in $CDCl_3$ (300 MHz).

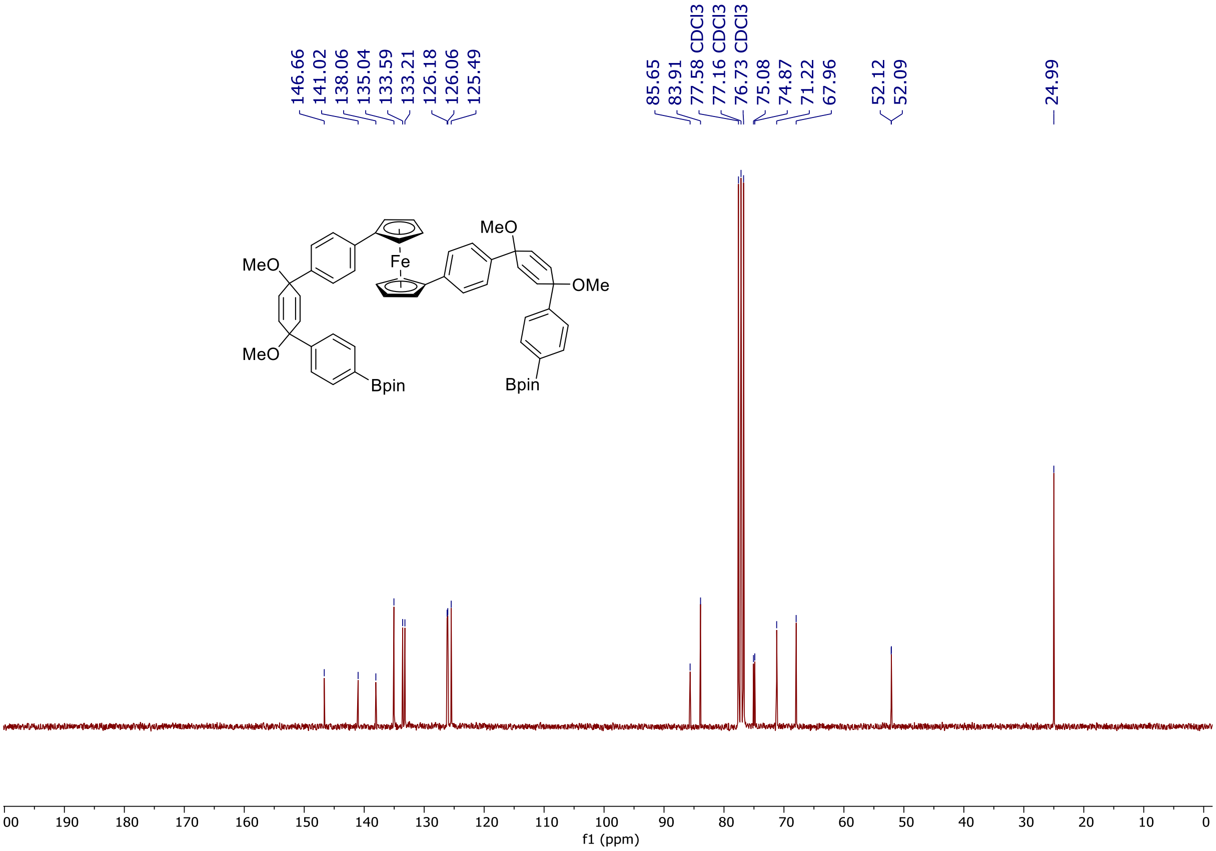

^{13}C NMR spectrum of compound **3** in $CDCl_3$ (101 MHz).

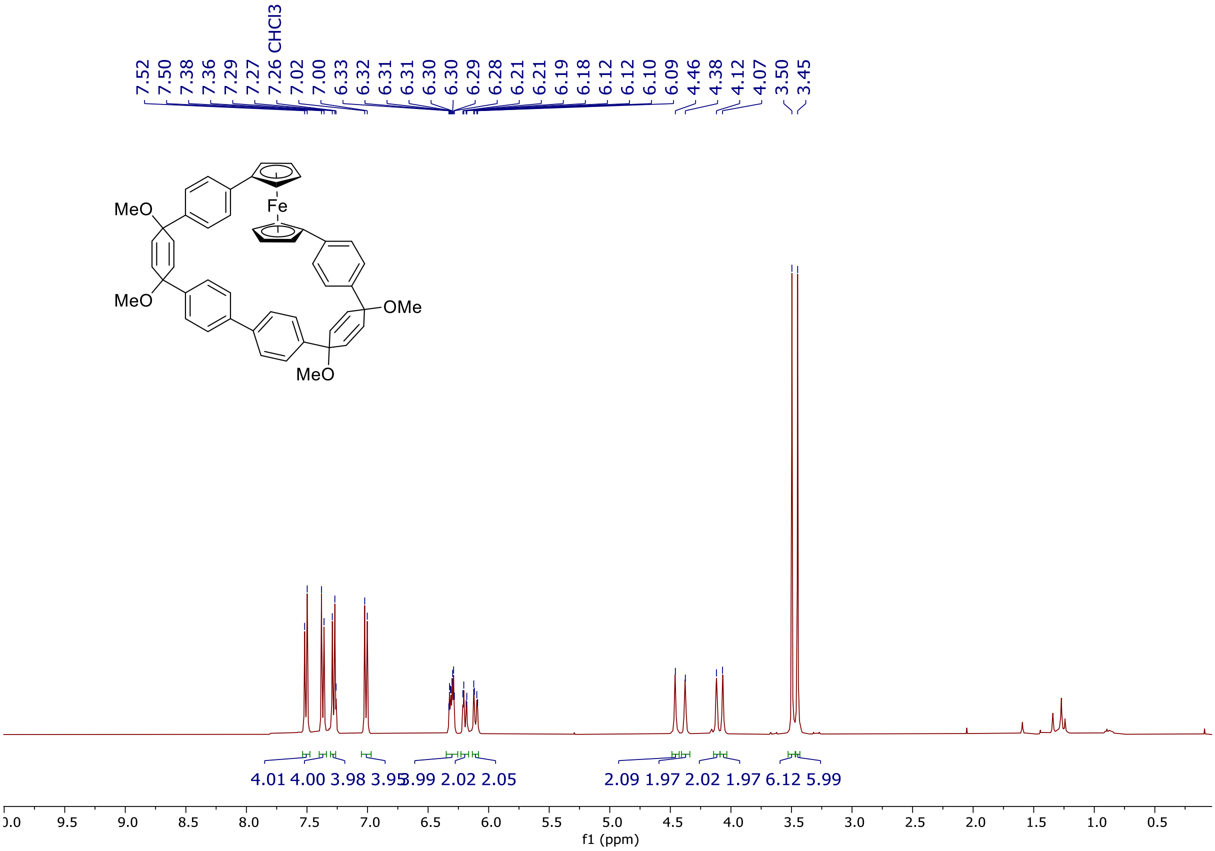

^{1}H NMR spectrum of compound **pro-Fc[6]CPP** in $CDCl_3$ (300 MHz).

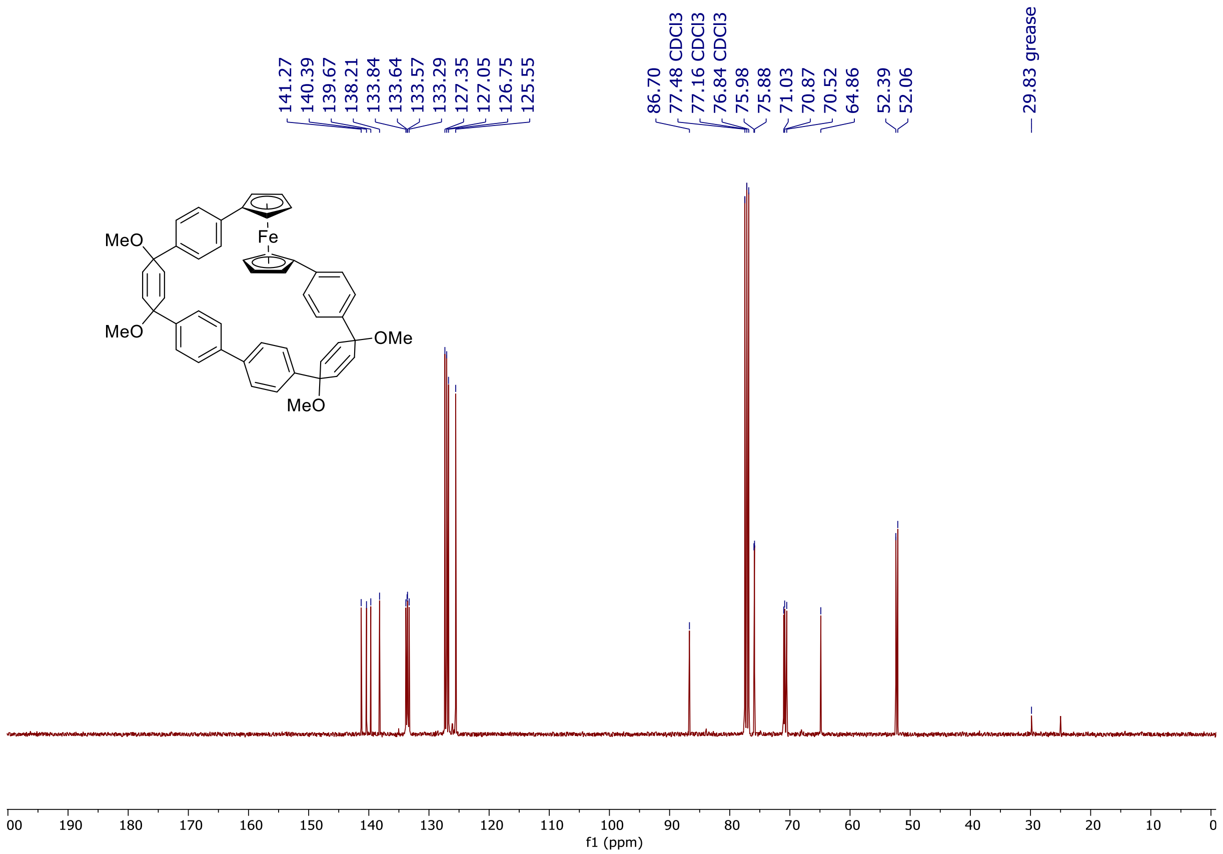

^{13}C NMR spectrum of compound **pro-Fc[6]CPP** in $CDCl_3$ (101 MHz).

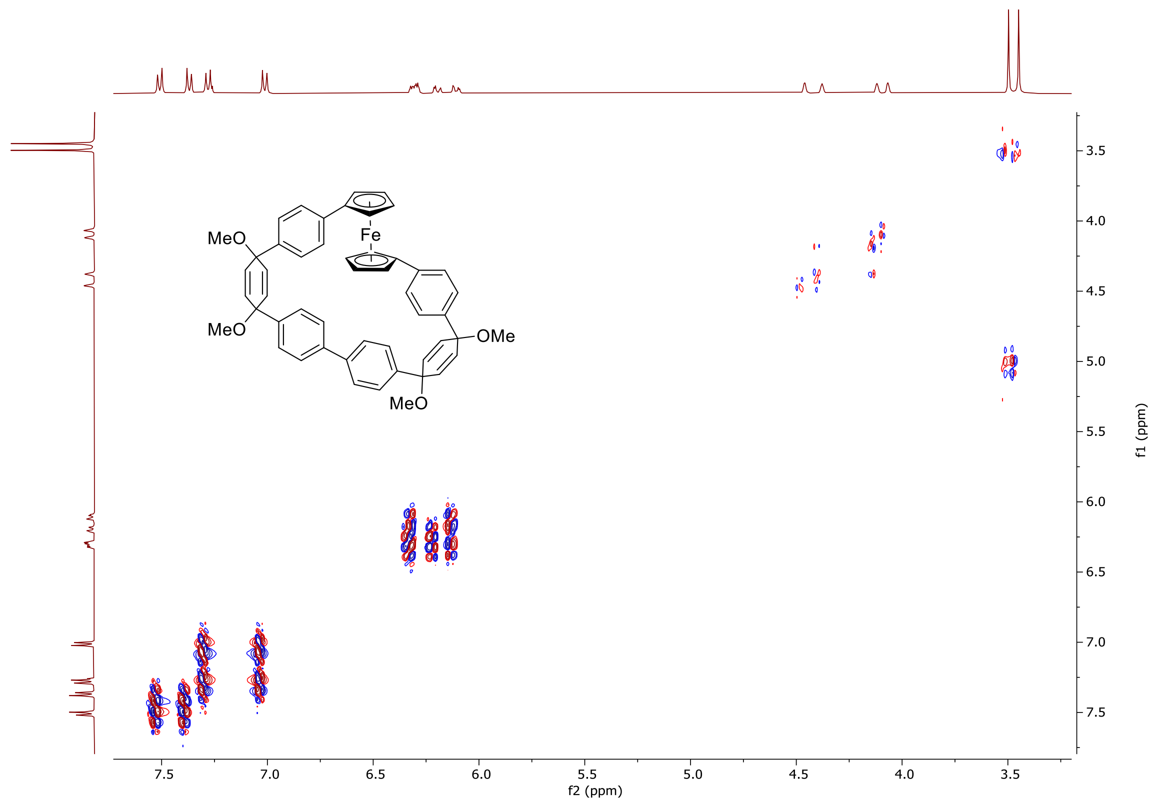

^{1}H–^{1}H COSY spectrum of compound **pro-Fc[6]CPP** in $CDCl_3$ (300 MHz).

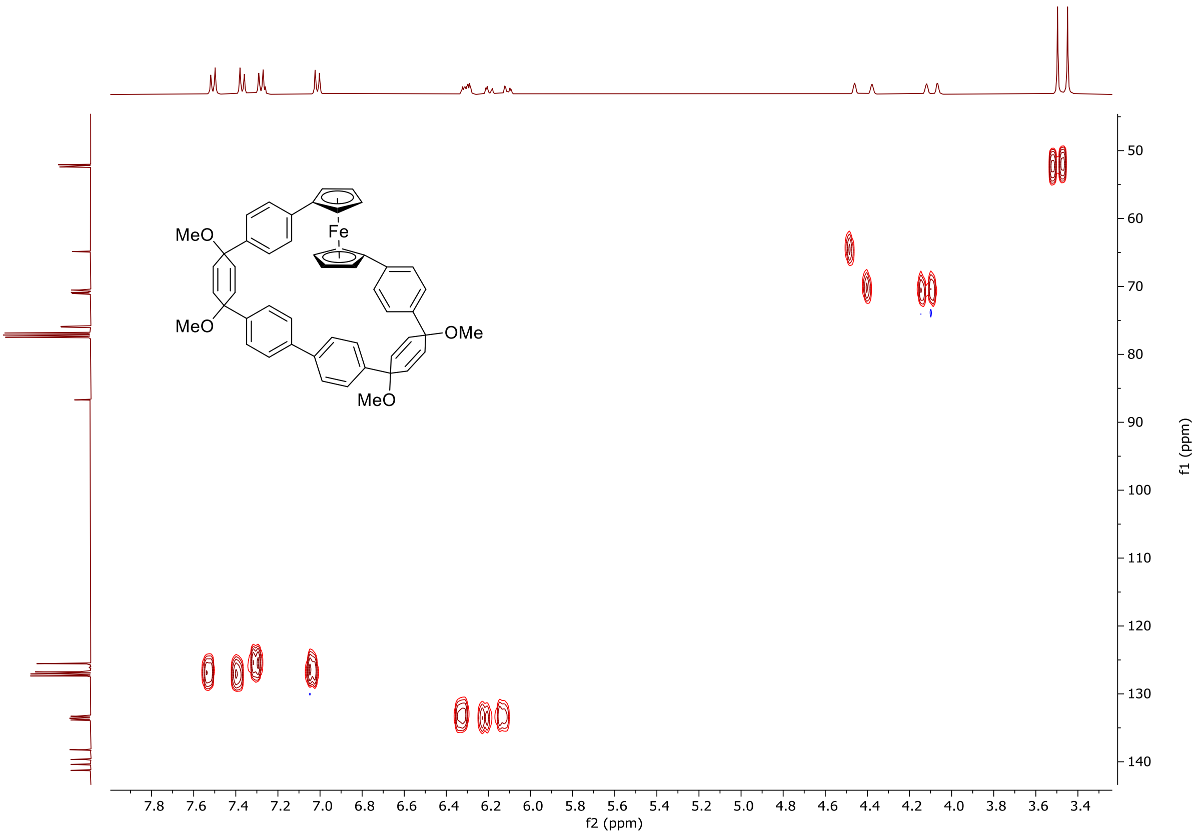

^{1}H– ^{13}C HSQC spectrum of compound **pro-Fc[6]CPP** in $CDCl_3$ (300/101 MHz).

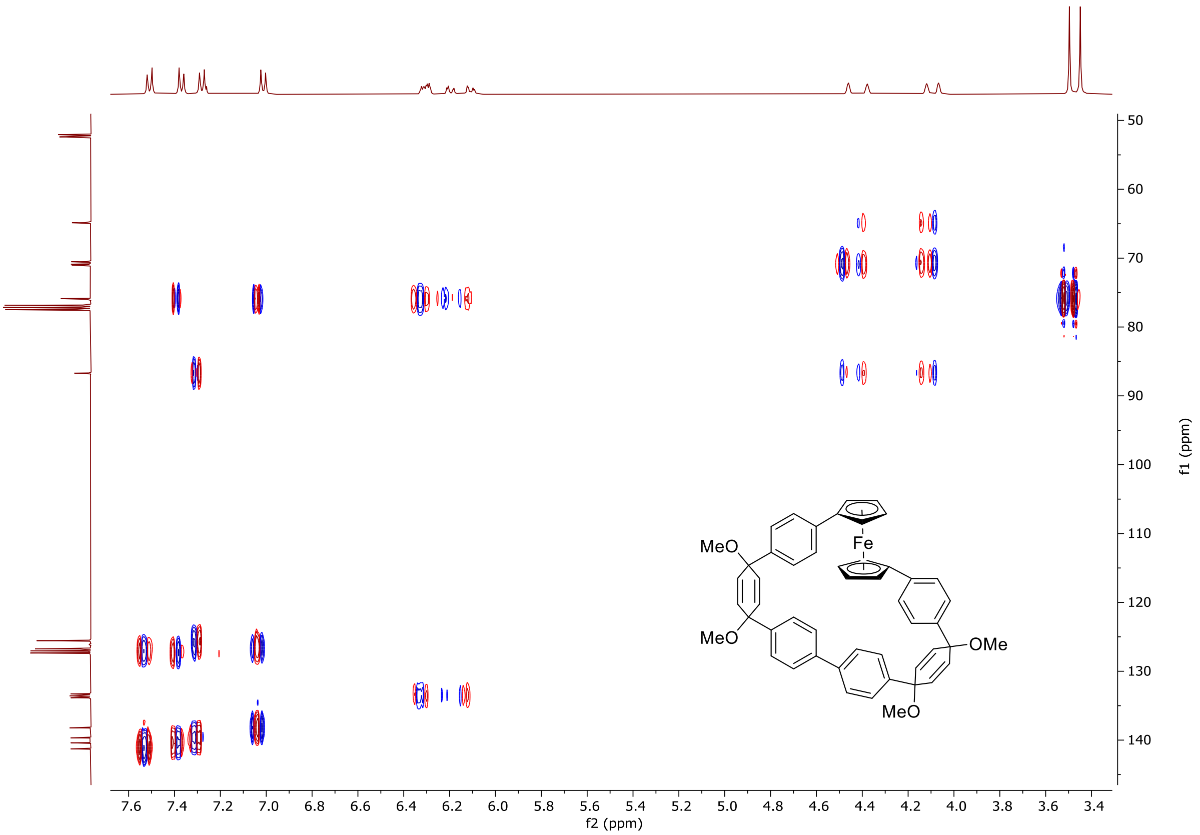

^{1}H– ^{13}C HMBC spectrum of compound **pro-Fc[6]CPP** in $CDCl_3$ (300/101 MHz).

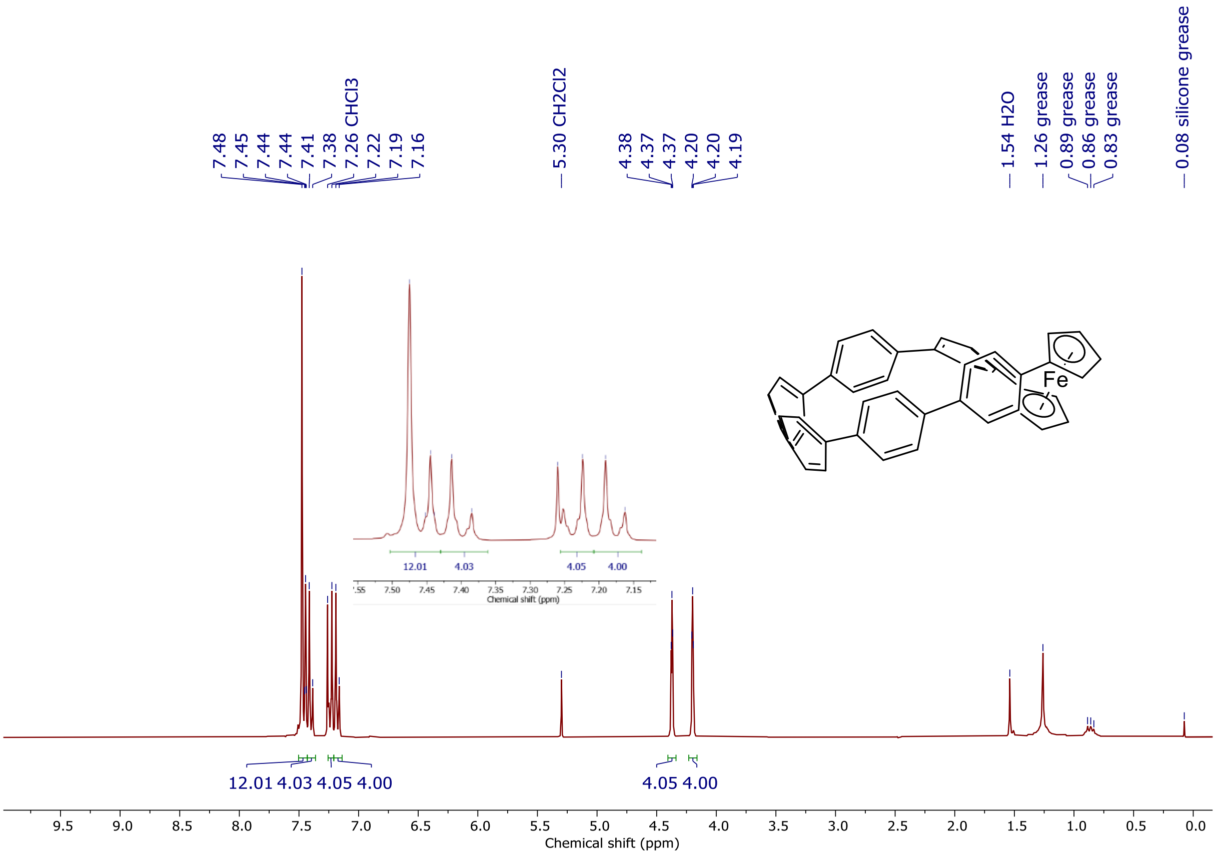

^{1}H NMR spectrum of compound **Fc[6]CPP** in $CDCl_3$ (300 MHz).

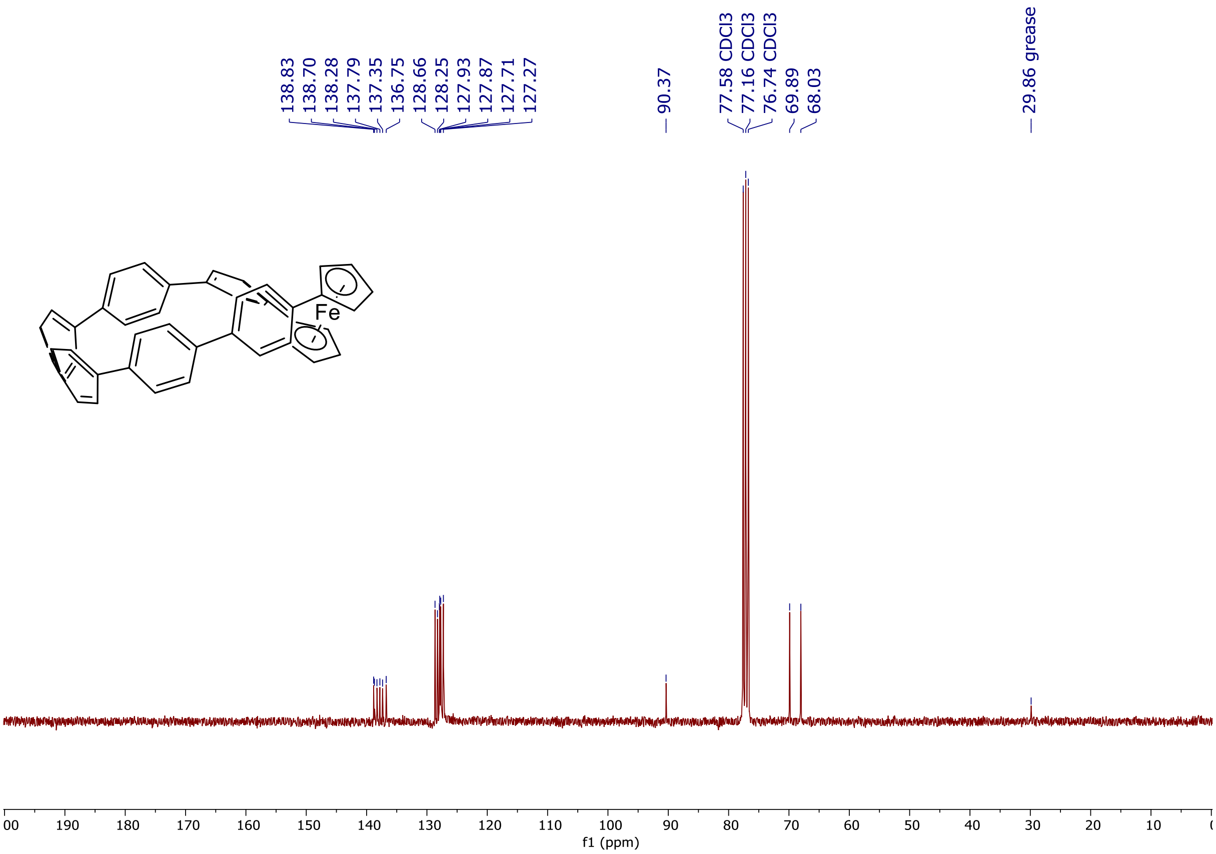

^{13}C NMR spectrum of compound **Fc[6]CPP** in $CDCl_3$ (75 MHz).

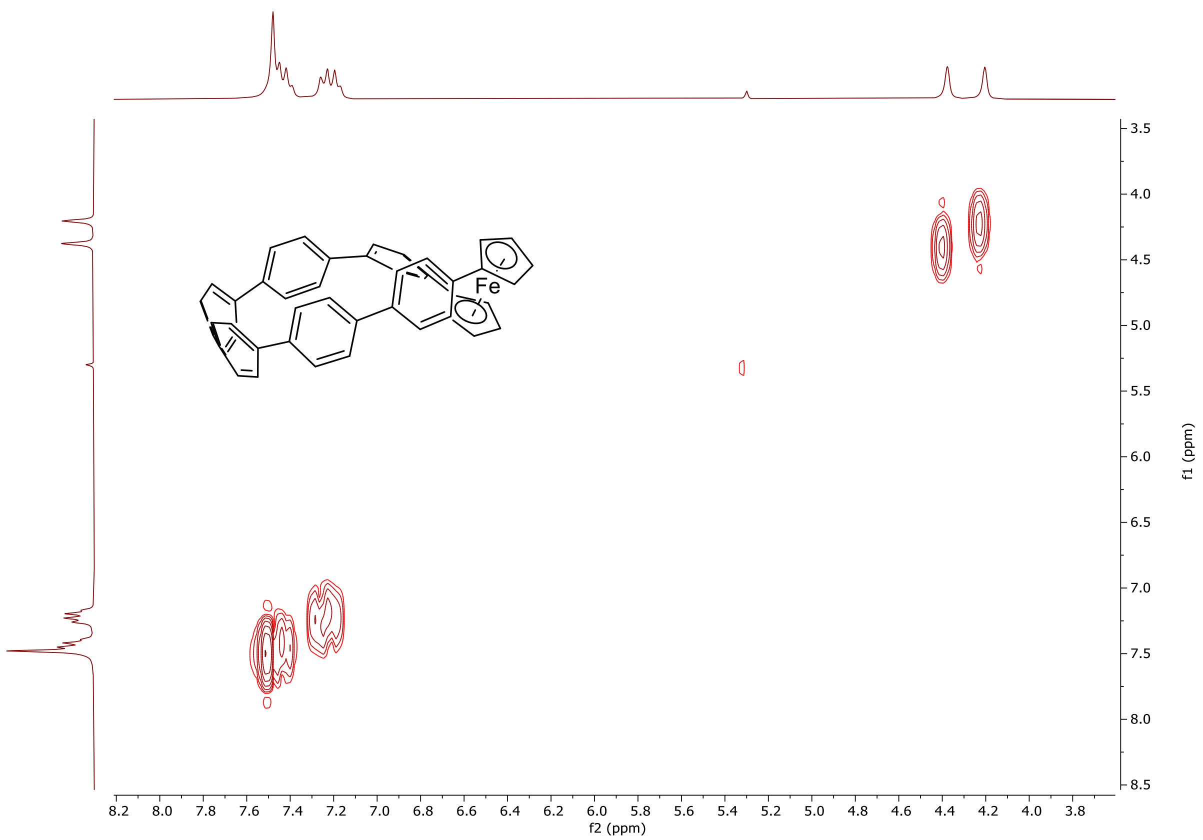

$^{1}H–^{1}H$ COSY spectrum of compound **Fc[6]CPP** in $CDCl_3$ (300 MHz).

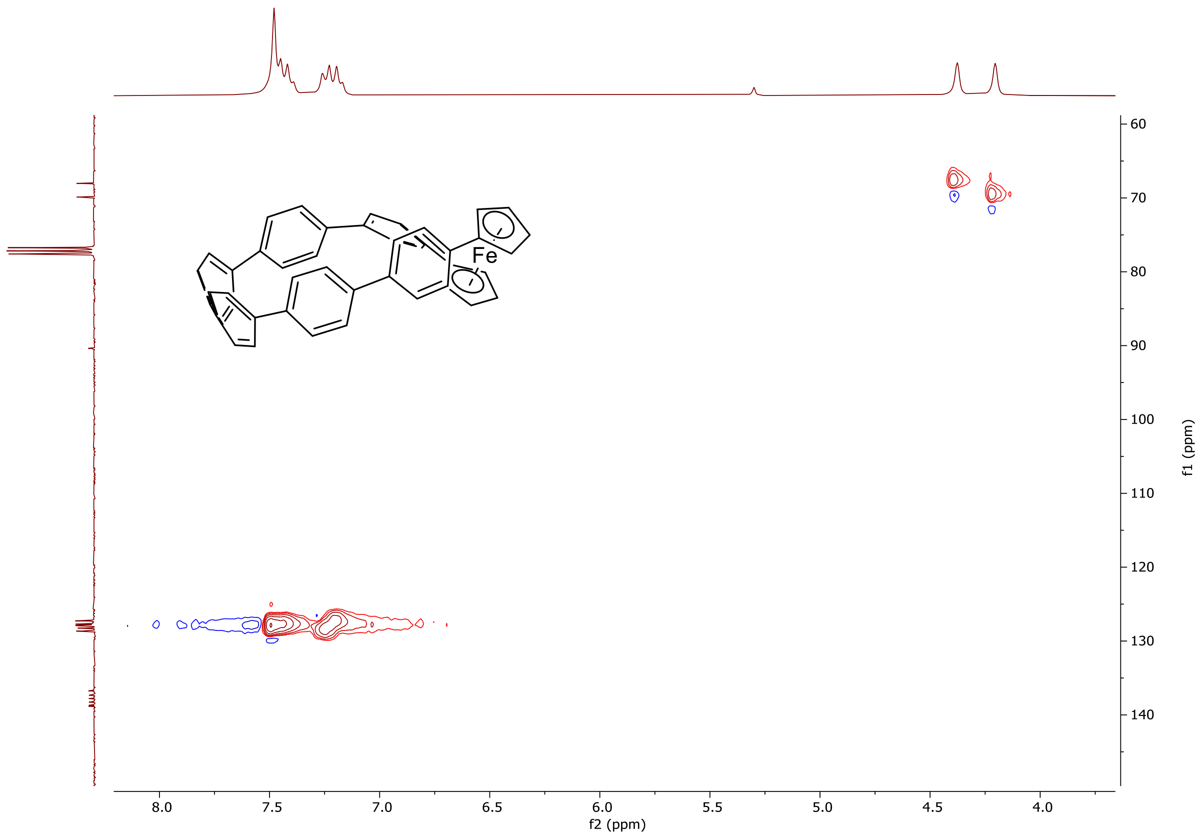

$^{1}H–^{13}C$ HSQC spectrum of compound **Fc[6]CPP** in $CDCl_3$ (300/75 MHz).

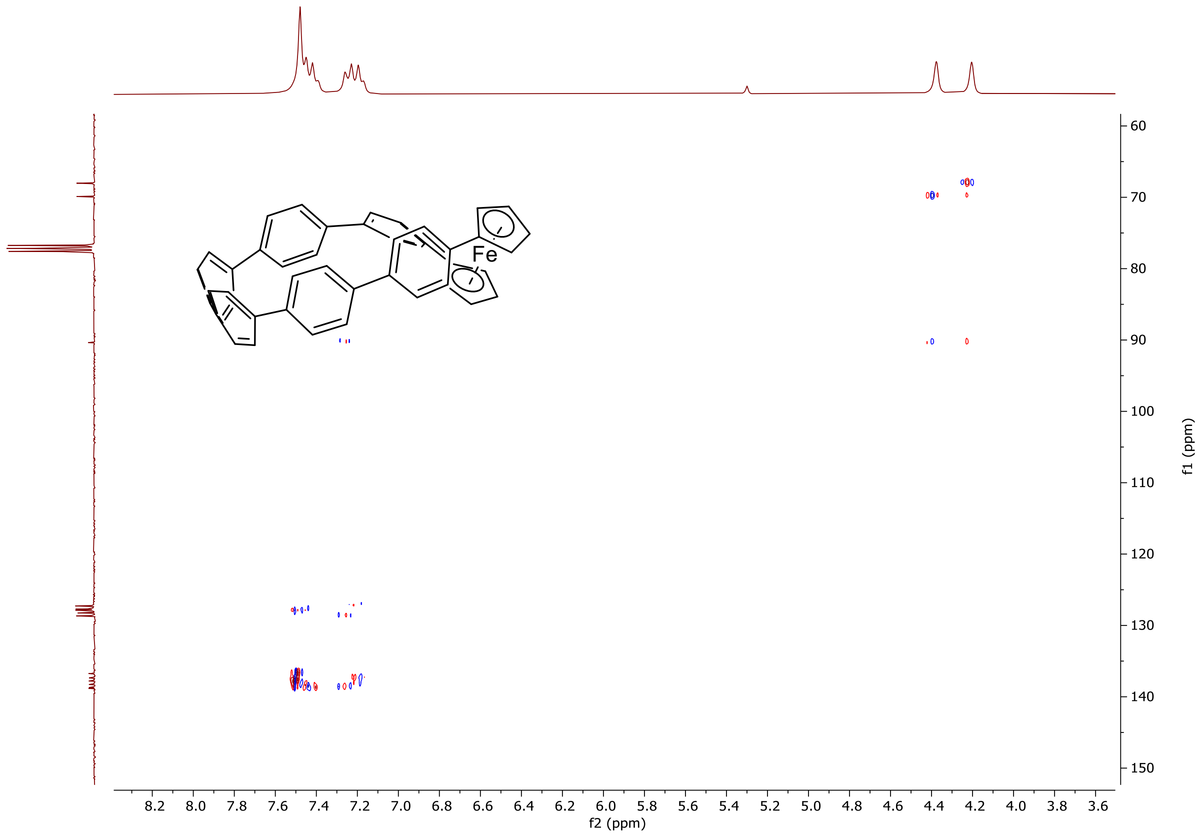

^{1}H– ^{13}C HMBC spectrum of compound **Fc[6]CPP** in CDCl3 (300/75 MHz).

8. Mass spectra

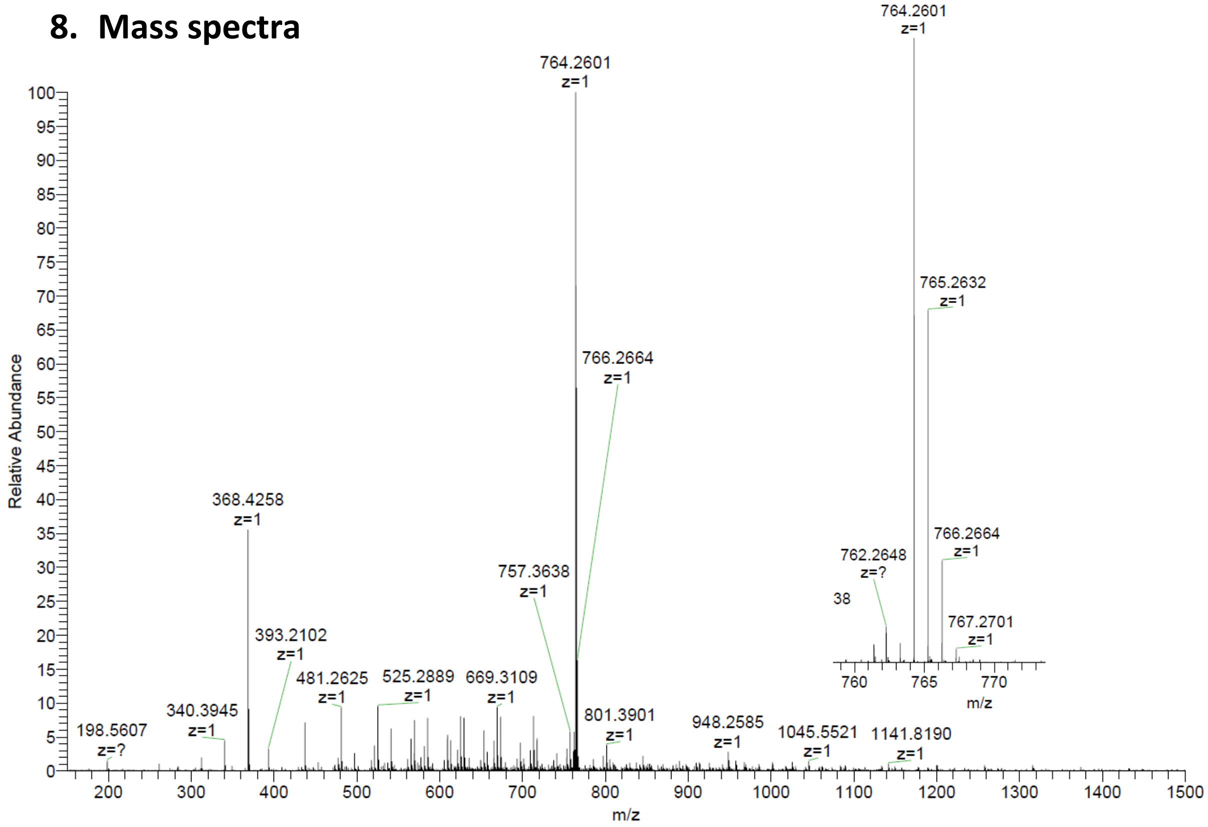

HRMS spectrum of compound **pro-Fc[6]CPP** (NSI pos. EtOAc).

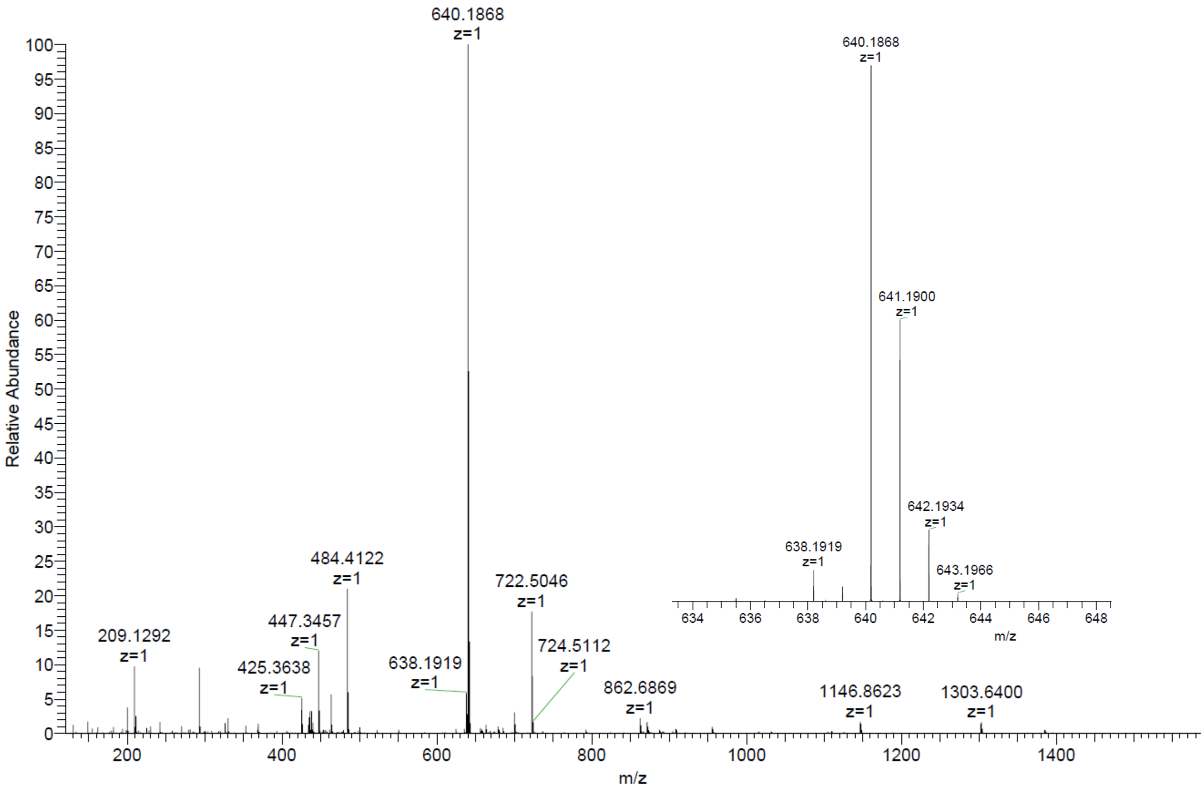

HRMS spectrum of compound **Fc[6]CPP** (NSI pos. EtOAc).

9. References

(1) Kleoff, M.; Schwan, J.; Boeser, L.; Hartmayer, B.; Christmann, M.; Sarkar, B.; Heretsch, P. Scalable Synthesis of Functionalized Ferrocenyl Azides and Amines Enabled by Flow Chemistry. *Org. Lett.* **2020**, *22* (3), 902–907. https://doi.org/10.1021/acs.orglett.9b04450.

(2) Sisto, T. J.; Golder, M. R.; Hirst, E. S.; Jasti, R. Selective Synthesis of Strained [7]Cycloparaphenylene: An Orange-Emitting Fluorophore. *J. Am. Chem. Soc.* **2011**, *133* (40), 15800–15802. https://doi.org/10.1021/ja205606p.

(3) Darzi, E. R.; White, B. M.; Loventhal, L. K.; Zakharov, L. N.; Jasti, R. An Operationally Simple and Mild Oxidative Homocoupling of Aryl Boronic Esters To Access Conformationally Constrained Macrocycles. *J. Am. Chem. Soc.* **2017**, *139* (8), 3106–3114. https://doi.org/10.1021/jacs.6b12658.

(4) Bruker, SAINT V8.40B, Bruker AXS Inc., Madison, Wisconsin, USA, 2001.

(5) Krause, L.; Herbst-Irmer, R.; Sheldrick, G. M.; Stalke, D. Comparison of Silver and Molybdenum Microfocus X-Ray Sources for Single-Crystal Structure Determination. *J. Appl. Crystallogr.* **2015**, *48* (1), 3–10. https://doi.org/10.1107/S1600576714022985.

(6) Sheldrick, G. M. *SHELXT* – Integrated Space-Group and Crystal-Structure Determination. *Acta Crystallogr. Sect. Found. Adv.* **2015**, *71* (1), 3–8. https://doi.org/10.1107/S2053273314026370.

(7) Spek, A. L. PLATON SQUEEZE: A Tool for the Calculation of the Disordered Solvent Contribution to the Calculated Structure Factors. *Acta Crystallogr. Sect. C Struct. Chem.* **2015**, *71* (1), 9–18. https://doi.org/10.1107/S2053229614024929.

(8) Spek, A. L. Single-Crystal Structure Validation with the Program PLATON. *J. Appl. Crystallogr.* **2003**, *36* (1), 7–13. https://doi.org/10.1107/S0021889802022112.

(9) Sheldrick, G. M. Crystal Structure Refinement with *SHELXL*. *Acta Crystallogr. Sect. C Struct. Chem.* **2015**, *71* (1), 3–8. https://doi.org/10.1107/S2053229614024218.
(10) *Electrochemical Methods: Fundamentals and Applications, 2nd Edition | Wiley*. https://www.wiley.com/en-us/Electrochemical+Methods%3A+Fundamentals+and+Applications%2C+2nd+Edition-p-9780471043720 (accessed 2024-07-29).
(11) Hong, Y. H.; Han, J. W.; Jung, J.; Nakagawa, T.; Lee, Y.-M.; Nam, W.; Fukuzumi, S. Photocatalytic Oxygenation Reactions with a Cobalt Porphyrin Complex Using Water as an Oxygen Source and Dioxygen as an Oxidant. *J. Am. Chem. Soc.* **2019**, *141* (23), 9155–9159. https://doi.org/10.1021/jacs.9b02864.
(12) Witzel, S.; Hoffmann, M.; Rudolph, M.; Rominger, F.; Dreuw, A.; Hashmi, A. S. K. A Radical Chain: Mononuclear “Gold Only” Photocatalysis. *Adv. Synth. Catal.* **2022**, *364* (3), 581–592. https://doi.org/10.1002/adsc.202101113.
(13) *Welcome to Prizmatix.com*. https://www.prizmatix.com/index.html (accessed 2024-08-06).
(14) Scuppa, S.; Orian, L.; Dini, D.; Santi, S.; Meneghetti, M. Nonlinear Absorption Properties and Excited State Dynamics of Ferrocene. *J. Phys. Chem. A* **2009**, *113* (33), 9286–9294. https://doi.org/10.1021/jp9047192.
(15) *Photo Sciences Publications | Kessil LED*. Kessil. https://www.Kessil.com/products/science_publications.php (accessed 2024-08-06).
(16) Frisch, M. J.; Trucks, G. W.; Schlegel, H. B.; Scuseria, G. E.; Robb, M. A.; Cheeseman, J. R.; Scalmani, G.; Barone, V.; Petersson, G. A.; Nakatsuji, H.; Li, X.; Caricato, M.; Marenich, A. V.; Bloino, J.; Janesko, B. G.; Gomperts, R.; Mennucci, B.; Hratchian, H. P.; Ortiz, J. V.; Izmaylov, A. F.; Sonnenberg, J. L.; Williams; Ding, F.; Lipparini, F.; Egidi, F.; Goings, J.; Peng, B.; Petrone, A.; Henderson, T.; Ranasinghe, D.; Zakrzewski, V. G.; Gao, J.; Rega, N.; Zheng, G.; Liang, W.; Hada, M.; Ehara, M.; Toyota, K.; Fukuda, R.; Hasegawa, J.; Ishida, M.; Nakajima, T.; Honda, Y.; Kitao, O.; Nakai, H.; Vreven, T.; Throssell, K.; Montgomery Jr., J. A.; Peralta, J. E.; Ogliaro, F.; Bearpark, M. J.; Heyd, J. J.; Brothers, E. N.; Kudin, K. N.; Staroverov, V. N.; Keith, T. A.; Kobayashi, R.; Normand, J.; Raghavachari, K.; Rendell, A. P.; Burant, J. C.; Iyengar, S. S.; Tomasi, J.; Cossi, M.; Millam, J. M.; Klene, M.; Adamo, C.; Cammi, R.; Ochterski, J. W.; Martin, R. L.; Morokuma, K.; Farkas, O.; Foresman, J. B.; Fox, D. J. Gaussian 16 Rev. C.02, 2016.
